# eQTL Meta‐Analysis Reveals Conserved and Population‐Specific Regulatory Variation Underlying Nutritional Trait Evolution and Domestication in Tomato

**DOI:** 10.1002/advs.202519899

**Published:** 2026-05-14

**Authors:** Jiantao Zhao, Xin Wang, Jing Zhang, Ryan McQuinn, Je Min Lee, Itay Gonda, Ari Feder, Yimin Xu, Baike Wang, Qinghui Yu, Denise M. Tieman, Sanwen Huang, Harry Klee, James J. Giovannoni, Zhangjun Fei

**Affiliations:** ^1^ Key Laboratory of Genome Research and Genetic Improvement of Xinjiang Characteristic Fruits and Vegetables Institute of Fruits and Vegetables Xinjiang Academy of Agricultural Sciences Urumqi Xinjiang China; ^2^ Boyce Thompson Institute Cornell University Ithaca New York USA; ^3^ National Key Laboratory for Germplasm Innovation & Utilization of Horticultural Crops Hubei Hongshan Laboratory College of Horticulture and Forestry Sciences Huazhong Agricultural University Wuhan China; ^4^ College of Horticulture Northwest A&F University Yangling Shaanxi China; ^5^ Horticultural Sciences University of Florida Gainesville Florida USA; ^6^ National Key Laboratory of Tropical Crop Breeding Shenzhen Branch Guangdong Laboratory of Lingnan Modern Agriculture Genome Analysis Laboratory of the Ministry of Agriculture and Rural Affairs Agricultural Genomics Institute at Shenzhen Chinese Academy of Agricultural Sciences Shenzhen Guangdong China; ^7^ U.S. Department of Agriculture‐Agricultural Research Service Robert W. Holley Center for Agriculture and Health Ithaca New York USA

**Keywords:** eQTL, fruit nutrition, meta‐analysis, tomato

## Abstract

Deciphering how genetic variation shapes transcriptional regulation is key to understanding the regulatory architecture of complex crop traits. Here, we present a comprehensive meta‐analysis of expression quantitative trait loci (eQTLs) across five diverse tomato populations. We construct a genome‐wide atlas of 40 928 eQTLs, comprising 9463 *cis‐* and 31 465 *trans‐*eQTLs, and reveal thousands of loci detectable only through meta‐analysis. A substantial fraction of these eQTLs is associated with transcription factors (TFs), including a co‐regulated cluster of two TFs and one transcriptional regulator targeted by a shared *trans*‐eQTL hotspot harboring a key enzyme gene in gibberellin biosynthesis. Genes underlying nutritional quality traits, including sugars, organic acids, carotenoids, and folates, are predominantly controlled by strong *cis*‐eQTLs. We further delineate a *MYB12*‐centered regulatory network coordinating flavonoid metabolism that also extends to folate biosynthesis. Remarkably, MYB12 functions as a *trans*‐regulator linking flavonoid and folate pathways, contributing to the reduced folate content characteristic of pink‐fruited tomatoes. Together, this eQTL meta‐analysis provides a high‐resolution regulatory map of transcriptional variation in tomato and highlights key regulators of fruit nutritional quality, providing biological insights and practical targets for crop improvement.

## Graphical abstract

A comprehensive meta‐analysis of expression quantitative trait loci (eQTLs) across five diverse tomato populations reveals a high‐resolution atlas of transcriptional regulation and uncovers conserved and population‐specific regulatory architectures underlying fruit nutritional quality traits, including flavonoids, sugars, organic acids, carotenoids, and folates. This study also highlights a novel role for the MYB12 transcription factor in regulating folate metabolism.

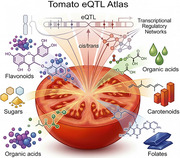

## 1 Introduction

Variation in gene expression plays a fundamental role in shaping plant development, metabolism, and adaptive responses to environmental and evolutionary pressures. Expression quantitative trait locus (eQTL) mapping has emerged as a powerful approach to dissect the genetic basis of transcriptional variation and to identify putative regulatory variants linked to key biological processes. eQTLs are broadly classified into *cis*‐eQTLs, which are located proximal to the genes they regulate, and *trans*‐eQTLs, which modulate gene expression from distant loci or even different chromosomes [1]. eQTL studies have provided valuable insights into the genetic architecture of gene expression and the role of regulatory variants in shaping key agronomic traits in major crops [2, 3, 4, 5, 6, 7]. They also serve as a crucial step for gene prioritization and effective prediction of gene functions [8]. However, most existing eQTL analyses have been limited to single populations, constraining their resolution and generalizability in dissecting the genetic architecture of complex traits such as yield and quality.

Meta‐analysis of genome‐wide association studies (GWAS) has proven effective in increasing statistical power and resolving population‐specific heterogeneity by integrating data across multiple studies [9]. GWAS meta‐analyses have been recently successfully applied in tomato [10] and rice [11], highlighting their great potential in identifying novel trait‐associated loci. In humans, eQTL meta‐analyses have markedly improved the identification of regulatory variants and the fine‐mapping of causal loci underlying complex traits [8, 12, 13]. However, such integrative eQTL meta‐analysis approaches remain rare in plants, particularly across multiple genetically diverse populations.

Tomato (*Solanum lycopersicum*), a globally cultivated fruit crop and a model system for studying fleshy fruit biology, exhibits striking natural variation in fruit color, flavor, and nutrient content, particularly between wild and cultivated accessions. These traits are driven by complex, multilayered transcriptional regulation, especially during fruit development and ripening, underpinning evolutionarily divergent genetic regulation of key metabolic pathways [14, 15, 16, 17]. In particular, the transcriptional regulation of metabolic pathways, including those involved in the biosynthesis of carotenoids, flavonoids, sugars, organic acids, and folates, directly influences tomato fruit appearance, nutritional value, and consumer appeal [3, 4, 5, 17]. For instance, the MYB transcription factor (TF) MYB12 (Solyc01g079620) acts as a master regulator of flavonoid biosynthesis. Mutations in MYB12 result in flavonoid deficiency, leading to a characteristic pink fruit phenotype due to the absence of yellow peel pigmentation [3, 18, 19, 20]. Several genes in the flavonoid biosynthetic pathway have been identified as direct *trans*‐regulatory targets of MYB12 through ChIP‐seq analyses [21]. However, a comprehensive understanding of how these pathways are genetically regulated across evolutionary divergent tomato populations and whether additional TFs or hormone‐regulating genes contribute to this regulation remains lacking.

Here, we present a large‐scale eQTL meta‐analysis that integrates transcriptomic and genomic data across five evolutionary and genetically distinct tomato populations. These include introgression lines (ILs) derived from crosses between cultivated tomatoes and the wild species *S. lycopersicoides* and *S. pennellii*; recombinant inbred lines (RILs) from crosses with *S. pimpinellifolium* and *S. cheesmaniae*; and a natural population. By integrating eQTL mapping across these diverse populations, our meta‐analysis captures broad allelic diversity and population‐specific genetic variation, enabling the identification of over 40,000 eQTLs, including 9463 *cis*‐eQTLs and 31 465 *trans*‐eQTLs. We identified both conserved and population‐specific *cis*‐ and *trans*‐regulatory eQTLs that control the expression of genes associated with key nutritional traits, including flavonoids, carotenoids, sugars, organic acids, and folates. Our findings uncover the regulatory architecture underlying tomato fruit nutritional quality and demonstrate the power of eQTL meta‐analysis in resolving transcriptional variation shaped by evolution, domestication, and selection. This work lays a foundation for the precision breeding of tomatoes with improved nutritional value to meet diverse consumer preferences.

## 2 Results

### 2.1 eQTL Mapping in Diverse Tomato Populations

To systematically dissect the genetic regulation of gene expression in tomato, we profiled transcriptomes across five diverse tomato populations: two interspecific introgression line (IL) populations derived from crosses *S. lycopersicum* × *S. lycopersicoides* (Lycop) [22] and *S. lycopersicum* × *S. pennellii* (Penne) [23]; two recombinant inbred line (RIL) populations from *S. lycopersicum* × *S. cheesmaniae* (Chees) [24] and *S. lycopersicum* × *S. pimpinellifolium* (Pimpi) [25]; and a natural diversity panel [3] consisting of *S. pimpinellifolium*, *S. lycopersicum* var. *cerasiforme*, and S. *lycopersicum* var. *lycopersicum* accessions (Table 1). Phenotypic variation between the parental lines has been extensively characterized, including traits such as fruit size, fruit color, carotenoid content, sugar content (Brix), and biotic/abiotic stress tolerance (Table S1). Together, these populations span a broad spectrum of the tomato gene pool, from deeply diverged wild species to cultivated backgrounds, enabling comprehensive discovery of both conserved and lineage‐specific eQTLs.

**TABLE 1:** Experiment design and summary statistics of eQTLs.

**Population**	**Code**	**Type**	**Parents**	**No. lines**	**No. genes expressed**	**No. SNPs**	**No. eQTLs**		
							**Total**	***Cis***	***Trans***
*S. lycopersicoides*	Lycop	IL	VF36 × LA2951	106	17 700	223 712	12 795	1356	11 439
*S. pennellii*	Penne	IL	M82 × LA716	76	18 411	292 081	3609	468	3141
*S. cheesmaniae*	Chees	RIL	UC204 × LA483	59	15 675	146 377	4255	652	3603
*S. pimpillifolium*	Pimpi	RIL	NC EBR‐1 × LA2093	136	18 065	185 241	20 427	6067	14 360
*S. pimpinellifolium*, *S. lycopersicum* var. *cerasiforme*, *S. lycopersicum* var. *lycopersicum*	Nature	Natural	—	379	19 965	2 040 403	16 201	4301	11 900

**FIGURE 1:**
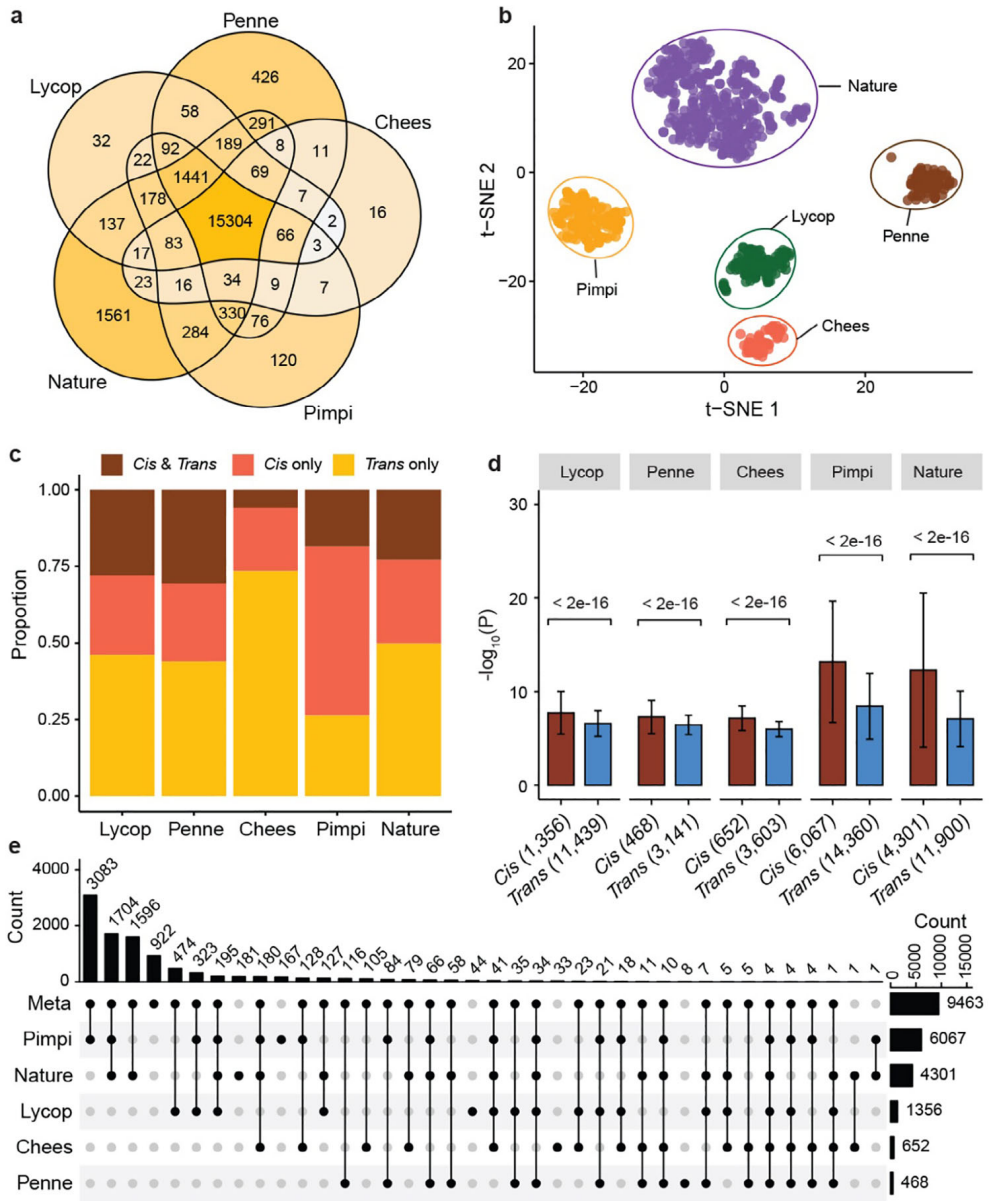
Population‐scale expression diversity and *cis*/*trans*‐regulatory architecture across different tomato populations. (a) Venn diagram showing the numbers of expressed genes across different populations. (b) t‐SNE plot of global gene expression profiles in different populations. (c) Proportions of genes associated with *cis‐*eQTLs only, both *cis‐* and *trans‐*eQTLs, or *trans‐*eQTLs only. (d) Significance levels (‐log_10_(P)) for *cis*‐ versus *trans*‐eQTLs across different populations. Data are presented as bar plots showing mean ± SD. *P*‐values were calculated using the Wilcoxon test. Sample sizes (n) are indicated in parentheses after each cis/trans regulatory effect. (e) UpSet plot of the numbers of genes associated with *cis*‐eQTLs detected in different populations and the meta‐eQTL analysis.

We conducted transcriptome profiling using RNA‐Seq for red ripe fruits of 106, 76, 59, and 136 lines from the Lycop, Penne, Chees, and Pimpi populations, respectively (Table S2). RNA‐Seq data from orange‐stage fruits of the natural diversity panel, comprising 379 accessions, were obtained from a previous study [3]. The number of expressed genes was relatively consistent across populations, ranging from 15 675 in the Chees RIL population to 19 965 in the natural population (Table 1), with most genes expressed across all populations (Figure 1a). To assess global transcriptome dynamics, we applied t‐distributed Stochastic Neighbor Embedding (t‐SNE) to log2‐transformed expression data, which revealed clearly separated clusters corresponding to each population, indicative of population‐specific transcriptional profiles shaped by genetic background (Figure 1b). Among these, the natural population, as expected, clearly showed the highest within‐group variation.

Using high‐resolution SNP data, we performed *cis‐* (<50 kb) and *trans‐* (≥50 kb or located on different chromosomes) eQTL mapping across the five populations. The number of detected eQTLs varied considerably, ranging from 3609 in the Penne ILs to 20 427 in the Pimpi RILs (Table 1). In all populations, *trans‐*eQTLs consistently outnumbered *cis‐*eQTLs. Notably, although the natural diversity panel had the highest SNP density, it exhibited only moderate numbers of *cis‐* (4301) and *trans‐* (11 900) eQTLs, likely due to the complexity of association mapping in structured populations, where complex relationships and unmodeled cofounder factors can decrease statistical power and increase noise.

Notably, distinct patterns of regulatory partitioning were observed across populations. The proportions of genes regulated by *cis*‐eQTLs only, by both *cis*‐ and *trans*‐eQTLs, and by *trans*‐eQTLs only were comparable in the Lycop and Penne IL populations. In contrast, in Chees, only about 6% of genes were regulated by both *cis‐* and *trans*‐eQTLs, while genes regulated by *trans*‐eQTLs only dominated (73.5%). In Pimpi, 55% of genes were regulated by *cis*‐eQTLs only, the highest proportion among all populations (Figure 1c; Figure S1). This enrichment of local regulatory control may be due to recent divergence or domestication‐driven selection that may preserve *cis*‐acting polymorphisms. Across all populations, *cis*‐eQTLs were generally more significantly associated with gene expression than *trans*‐eQTLs, especially in the Pimpi RIL and the natural populations (Figure 1d). Collectively, these findings highlight the intricate complexity of transcriptional regulation in the Solanum clade and underscore the power of leveraging genetically diverse populations for regulatory genomics in tomato.

### 2.2 eQTL Meta‐Analysis

To comprehensively dissect the genetic architecture underlying gene expression variation, we conducted a meta‐analysis by integrating eQTLs identified across the five populations. This integrative approach leveraged both high mapping resolution and broad allelic diversity, enabling the discovery of regulatory variants with increased statistical power and cross‐population robustness. The meta‐analysis identified a total of 40 928 eQTLs, including 9463 *cis‐*eQTLs and 31 465 *trans‐*eQTLs, associated with 12 514 genes (Table S3), substantially more than the eQTLs detected in any individual population. Among these, 922 *cis*‐eQTLs and 7688 *trans*‐eQTLs‒associated with 1128 genes‒were uniquely identified through the meta‐analysis, underscoring the added power of meta‐analysis (Figure 1e; Figure S2). The identification of more *trans*‐eQTLs than *cis*‐eQTLs has been commonly observed in tomato and other species [3, 4, 5, 6, 26]. Among genes with *cis*‐eQTLs, 5259 showed associations exclusively with *cis‐*eQTLs. In these cases, the Pimpi population showed the strongest similarity to the meta‐eQTL results, followed by the natural population (Figure 1e). This high concordance likely reflects the genetic proximity of S. pimpinellifolium to the cultivated tomato gene pool and the relatively large sample size, both of which could enhance the detection of conserved *cis*‐regulatory variants. A total of 4204 genes were associated with both *cis‐* and *trans‐*eQTLs, involving 27 655 *trans‐*eQTLs, while 3051 genes were associated exclusively with 8014 *trans‐*eQTLs. For all *trans*‐eQTL‐associated genes, the natural population showed the strongest similarity to the meta‐eQTL results, followed by the Pimpi population (Figure S2). These differential overlap patterns suggest that *trans*‐regulatory networks may be better captured in populations with broader genetic diversity and larger sample sizes.

To provide a global perspective on conserved regulation variation, we analyzed eQTLs identified in the meta‐analysis and detected in at least two individual populations. Genes associated with conserved *cis*‐eQTLs (3167 genes) and conserved *trans*‐eQTLs (430 genes) were predominantly detected in the Pimpi and natural diversity panels (Figure S3a,b). Gene Ontology (GO) enrichment analysis revealed that genes harboring conserved *cis*‐eQTLs were significantly overrepresented in core metabolic processes (Figure S3c), while no significant functional enrichment was identified in genes under conserved *trans*‐regulation. This pattern suggests that *cis*‐regulatory evolution may be more constrained, consistent with recent studies [27, 28]. Although we did not observe major differences in the genomic distributions of genes associated with conserved and population‐specific *cis*‐eQTLs (Figure S4a), genes associated with conserved eQTLs exhibited significantly higher expression levels compared to those associated with population‐specific eQTLs (Figure S4b). We also noted that a substantial number of *trans*‐eQTLs detected in individual populations were not captured in the meta‐analysis, likely due to the more stringent genome‐wide significance cutoff used in the meta‐analysis to reduce false positives.

Our analysis revealed significant enrichment of eQTL associations among genes under selection during tomato domestication and improvement (Fisher's exact test, *P* < 0.001). Specifically, of the 5605 genes under domestication selection reported in a previous study [18], 1824 (32.6%) exhibited *cis*‐eQTL associations, and 1173 (20.9%) showed *trans*‐eQTL associations. Similarly, among the 4808 genes under improvement selection, 1452 (30.2%) and 1,016 (21.1%) were associated with *cis*‐ and *trans*‐eQTLs, respectively. Notably, genes under selection with eQTL associations displayed distinct expression characteristics compared with selected genes lacking eQTLs. For both domestication‐ and improvement‐selected genes, those harboring *cis*‐ or *trans*‐eQTLs exhibited significantly higher expression levels than selected genes without eQTLs (Wilcoxon rank‐sum test, *P* < 2 × 10^−16^; Figure S5). No significant difference in expression levels was observed between genes with *cis*‐ versus *trans*‐eQTLs, indicating that both regulatory mechanisms contribute comparably to expression divergence during selection. Collectively, these findings suggest that regulatory variation‒whether *cis* or *trans*‒is associated with enhanced expression changes in genes targeted by selection during tomato breeding.

Notably, the majority of *cis*‐eQTLs (n = 7962) were associated with peak SNPs located more than 2000 bp away from the target gene body, while only 1183 were located within gene bodies, 134 in proximal promoter regions (≤ 2000 bp upstream), and 184 in downstream regions (≤ 2000 bp). This distribution likely reflects the influence of linkage disequilibrium and suggests a prominent role of distal regulatory elements, such as enhancers or long‐range promoters, in *cis* regulation of gene expression. These findings underscore the importance of non‐coding genomic regions in shaping transcriptional diversity during tomato fruit development. Collectively, these results reveal a complex and diverse gene regulatory landscape and demonstrate that eQTL meta‐analysis can both uncover novel regulatory variants and increase confidence in previously identified associations, highlighting the value of integrating complementary populations in fine‐mapping robust and phylogenetically conserved regulatory loci associated with dynamic transcriptomic changes.

### 2.3 *Cis* and *Trans* Regulatory Landscape of Transcription Factors

**FIGURE 2:**
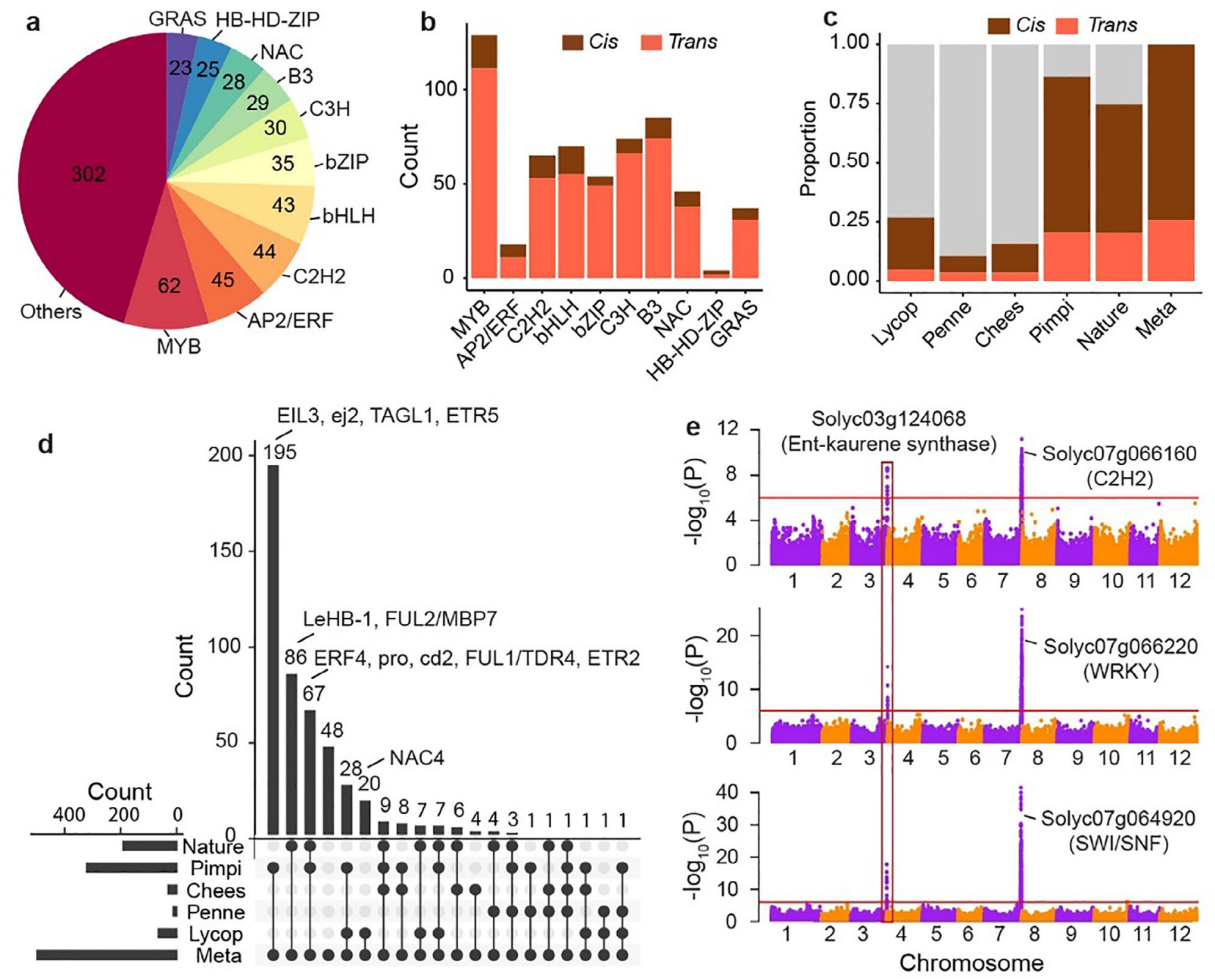
eQTL landscape of transcription factors. (a) Top ten transcription factor (TF) families associated with eQTLs in the meta‐analysis. (b) Numbers of *cis*‐ and *trans*‐eQTLs associated with the top ten TF families. (c) Proportions of *cis*‐ and *trans*‐eQTLs associated with TFs among all TF‐associated eQTLs detected in the meta‐analysis. (d) UpSet plot showing overlaps of TF‐associated *cis*‐eQTLs across populations. (e) Manhattan plots of the eQTL meta‐analysis for TFs *Solyc07g066160* (C2H2) and *Solyc07g066220* (WRKY), and transcriptional regulator *Solyc07g064920* (SWI/SNF), highlighting a shared *trans*‐eQTL (red rectangle box) harboring the gene *Solyc03g124068* (ent‐kaurene synthase).

Transcription factors (TFs), as core regulators of gene expression, play pivotal roles in tomato fruit development and ripening. Leveraging the power of eQTL meta‐analysis across diverse interspecific and natural populations, we detected a total of 1809 eQTLs associated with 666 TFs, including 498 *cis‐*eQTLs and 1311 *trans‐*eQTLs (Table S4). The most represented TF families included MYB, AP2/ERF, C2H2, bHLH, bZIP, C3H, B3, NAC, HB‐HD‐ZIP, and GRAS (Figure 2a), many of which have been implicated in hormone signaling pathways, chromatin remodeling, and fruit development. KEGG enrichment analysis of these transcription factors further supported their involvement in hormonal regulation, with “plant hormone signal transduction” identified as the most significantly enriched pathway (fold enrichment = 9.31; adjusted *P* = 3.98 ×10^−14^). Notably, TFs with both *cis‐* and *trans‐*eQTLs harbored, on average, about five times more *trans*‐eQTLs per TF family when compared with *cis*‐eQTLs (Figure 2b).

To assess the evolutionary conservation of regulatory relationships, we evaluated eQTL consistency across populations. Among TF‐associated eQTLs, 199 were shared by at least two populations, the majority of which (145; 72.9%) were *cis*‐eQTLs (Table S5), likely representing a core set of conserved TF‐associated eQTLs maintained across tomato divergence. The distribution of these conserved eQTLs varied markedly across populations: the Pimpi RIL population showed the highest number, followed by the natural diversity panel, whereas the more distantly related interspecific populations harbored far fewer conserved eQTLs (Figure 2c), suggesting substantial divergence of regulatory architectures with increasing phylogenetic distance from domesticated tomato. In addition to conserved regulatory elements, we identified population‐specific regulatory variants, including 86 and 195 *cis‐*eQTLs unique to the natural diversity panel and the Pimpi RIL population, respectively (Figure 2d). Notably, TFs associated with conserved eQTLs included previously reported key transcription factors regulating tomato fruit development, such as EIL3 [29], ej2 [30], and FUL2/MAP7 [31] (Figure 2d), reinforcing the physiological relevance of our eQTL meta‐analysis.

Strikingly, we identified a co‐regulated cluster comprising two TFs, *Solyc07g066160* (C2H2 zinc finger) and *Solyc07g066220* (WRKY), along with one transcriptional regulator, *Solyc07g064920* (SWI/SNF), all targeted by the same *trans‐*eQTL hotspot that harbored *Solyc03g124068*, which encodes ent‐kaurene synthase, a key enzyme in the gibberellin (GA) biosynthesis pathway (Figure 2e). Notably, we observed a strong positive correlation between the expression of *Solyc03g124068* and that of *Solyc07g066160*, *Solyc07g066220*, and *Solyc07g064920* (Figure S6). Collectively, these findings suggest a potential hormone‐responsive transcriptional module that integrates GA signaling with chromatin remodeling and stress‐responsive TFs to regulate tomato fruit ripening [32, 33] and highlight a complex and dynamically evolving regulatory landscape in tomato, in which TFs act both as targets and mediators of gene expression variation. Our results provide new avenues for dissecting and manipulating transcriptional networks that control fruit ripening and quality.

### 2.4 MYB12‐Centered Regulatory Network of Flavonoid Metabolism

**FIGURE 3:**
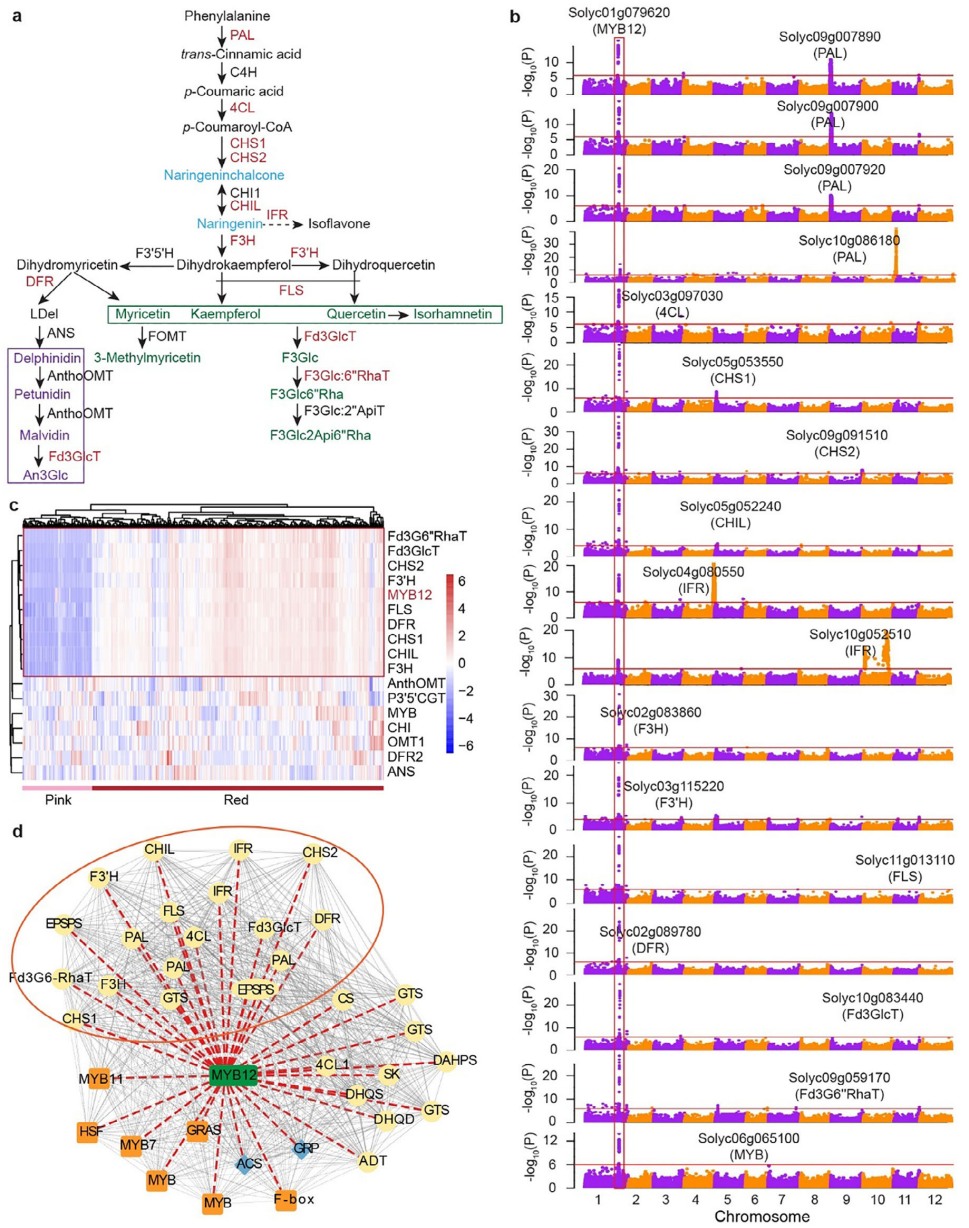
*MYB12*‐centered regulatory network of flavonoid metabolism in tomato. (a) Genes in the flavonoid biosynthesis and degradation pathway. Blue, green, and purple labels indicate flavanones, flavonols, and anthocyanins, respectively. Genes in red are associated with the *trans*‐eQTL harboring *MYB12*. PAL, phenylalanine ammonia‐lyase; 4CL, 4‐coumarate:CoA ligase; CHS1, chalcone synthase 1; CHS2, chalcone synthase 2; CHIL, chalcone‐flavonone isomerase; IFR, isoflavone reductase; F3H, flavanone 3‐hydroxylase; F3'H, flavonoid 3'‐monooxygenase; FLS, flavonol synthase; DFR, dihydroflavonol 4‐reductase; Fd3GlcT, flavonoid 3‐o‐glucosyltransferase; Fd3G6''RhaT, flavonoid‐3‐o‐glc‐6''‐o‐rhamnosyltransferase; MYB, MYB transcription factor; ANS, anthocyanidin synthase; AnthOMT, anthocyanin o‐methyltransferase. (b) Manhattan plots of eQTL meta‐analysis for flavonoid pathway genes, highlighting a *trans*‐eQTL hotspot associated with the majority of flavonoid pathway genes that harbors the transcription factor *MYB12* (*MYB12*‐*trans*‐eQTL). (c) Expression levels of flavonoid pathway genes associated (red box) or not associated with the *MYB12*‐*trans*‐eQTL. (d) Co‐expression module of *MYB12* and flavonoid pathway genes. Genes associated with the *MYB12*‐*trans*‐eQTL are indicated inside the orange circle. Genes highlighted in yellow, orange, and cyan represent flavonoid pathway genes, co‐expressed transcription factors, and hormone‐related signaling genes, respectively.

Flavonoids are important secondary metabolites in tomato, contributing to antioxidant activity, pigmentation, and nutritional value. To better understand the transcriptional regulation of flavonoid metabolism, we examined the eQTL profiles of core flavonoid biosynthesis and degradation pathway genes (Figure 3a). Notably, most flavonoid‐related genes were co‐associated with a single, highly significant *trans‐*eQTL hotspot (Figure 3b) that harbors *MYB12* (*Solyc01g079620*), a known regulator of flavonoid accumulation in tomato. *MYB12* is the causal gene for the *y* (yellow) mutation, which results in a colorless epidermis and pink fruit [3, 19, 20]. Several genes have already been validated as direct regulatory targets of *MYB12* through ChIP‐seq analysis [21]. This *MYB12*‐associated *trans‐*eQTL was uniquely identified in the natural diversity panel, the only population containing pink‐fruited accessions. Accessions carrying the alternative allele showed significantly lower expression of all associated target genes in the flavonoid biosynthetic pathway compared to those carrying the reference allele. Interestingly, accessions carrying heterozygous alleles exhibited significantly higher expression of these genes than those with reference homozygotes, suggesting a potential heterosis with possible implications for flavonoid breeding (Figure S7). Notably, in red fruits, flavonoid pathway genes linked to this *trans‐*eQTL exhibited substantially higher expression levels and showed strong positive correlations with *MYB12* transcript abundance, in contrast to pathway genes not associated with this regulatory locus (Figure 3c). Genes linked to this *trans‐*eQTL also exhibited significant differential expressions in peels between WT and *MYB12* mutant lines (Table S6). These findings further support the critical role of *MYB12* in regulating the transcription of flavonoid‐related genes and reveal the MYB12‐centered regulatory network in controlling flavonoid metabolism in tomato fruit.

The regulatory architecture was further supported by weighted gene co‐expression network analysis (WGCNA), which grouped most *MYB12*‐regulated flavonoid genes into the same co‐expression module (Figure 3d). Interestingly, this module also included five additional MYB transcription factors, including *Solyc06g065100*, which was also associated with the *MYB12 trans‐*eQTL. The presence of multiple co‐regulated MYB TFs suggests a hierarchical or combinatorial regulatory network in which MYBs reinforce or fine‐tune the flavonoid pathway activation. In addition to MYBs, the module also contained other regulatory genes such as an F‐box protein (*Solyc11g064780*), a GRAS‐domain transcription factor (*Solyc02g092370*), and a heat shock transcription factor (*Solyc12g007070*), suggesting crosstalk between stress responses, development, and secondary metabolism. Furthermore, two hormone‐related genes were identified in the module: *Solyc08g081550* (*ACS1A*), which encodes 1‐aminocyclopropane‐1‐carboxylate synthase, a key enzyme in ethylene biosynthesis, and *Solyc12g042500*, which encodes a gibberellin‐regulated protein. These findings suggest that *MYB12* may play a broader role in connecting flavonoid metabolism with additional TFs and hormone signaling pathways. GO enrichment analysis of the *MYB12* co‐expression module revealed significant overrepresentation of metabolic processes, particularly those related to organic and carboxylic acid metabolism (Figure S8a). Notably, the module was also significantly enriched for the biosynthesis of small molecules, including phenylpropanoids and carboxylic acids. KEGG pathway analysis further corroborated these findings, identifying “phenylpropanoid biosynthesis” as the most significantly enriched pathway, followed by “biosynthesis of amino acids” and “phenylalanine, tyrosine and tryptophan biosynthesis” (Figure S8b). These results collectively underscore the central role of *MYB12* in regulating the phenylpropanoid pathway and its integration with core metabolic networks.

### 2.5 *Cis*‐Regulatory Control of Carotenoid Pathway Genes

**FIGURE 4:**
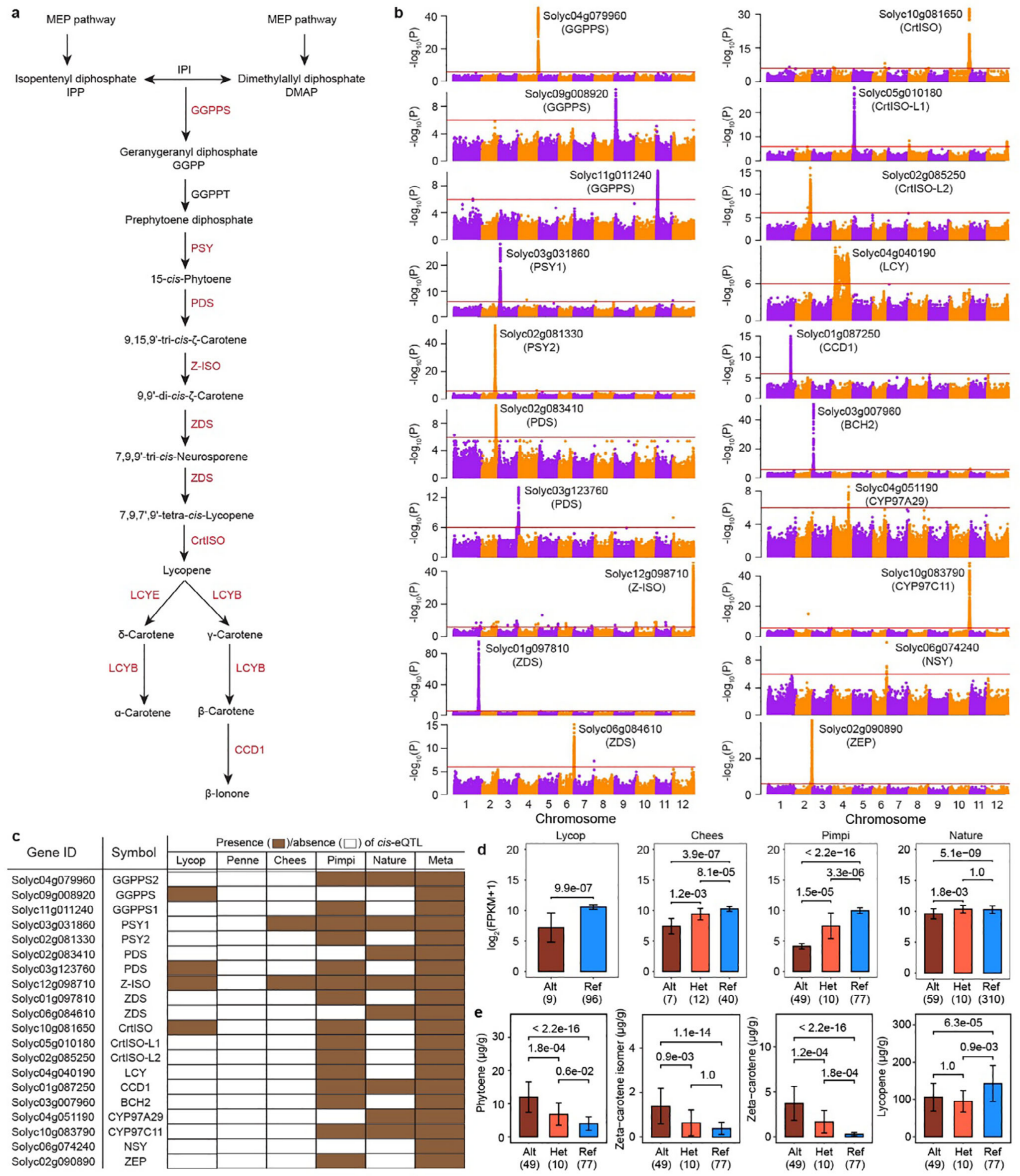
*Cis*‐regulation of carotenoid biosynthesis in tomato. (a) Diagram of the carotenoid biosynthetic pathway. IPI, isopentenyl diphosphate isomerase; GGPPS, geranylgeranyl diphosphate synthase; GGPPT, geranylgeranyl diphosphate transferase; PSY, phytoene synthase; PDS, phytoene desaturase; Z‐ISO, ζ‐carotene isomerase; ZDS, ζ‐carotene desaturase; CrtISO, carotenoid isomerase; LCYE, lycopene ε‐cyclase; LCYB, lycopene β‐cyclase; CCD1, carotenoid cleavage deoxygenase 1. (b) Manhattan plots of eQTL meta‐analysis for carotenoid biosynthetic genes, highlighting strong *cis*‐eQTL signals. LCY, lycopene cyclase; BCH2, β‐carotene hydroxylase 2; CYP97A29, cytochrome P450‐type monooxygenase 97A29; CYP97C11, cytochrome P450‐type monooxygenase 97C11; NSY, neoxanthin synthase; ZEP, zeaxanthin epoxidase. (c) Presence/absence of *cis*‐eQTLs associated with carotenoid biosynthetic genes across different populations. (d) Expression levels of *Z‐ISO* in accessions carrying the reference (Ref), alternative (Alt), or heterozygous (Het) alleles at the peak‐associated locus (Chr12:65720056; Chr, Chromosome) across populations. (e) Carotenoid contents in accessions carrying the Ref, Alt, or Het alleles at the peak‐associated locus of *Z‐ISO* in the Pimpi RIL population. Data in d and e are presented as bar plots showing mean ± SD. *P*‐values were calculated using the Wilcoxon test. Sample sizes (n) are indicated in parentheses below each genotype.

Carotenoids are important metabolites that determine fruit pigmentation, nutritional value, and consumer appeal in tomato. To uncover the genetic determinants underlying carotenoid metabolism, we examined eQTL profiles of carotenoid biosynthesis and degradation genes (Figure 4a). Notably, a substantial proportion of these genes were associated with single significant *cis*‐eQTLs (Figure 4b), suggesting that local regulatory variation plays a major role in controlling carotenoid gene expression in tomato.

Despite being distributed across different chromosomes, these carotenoid biosynthesis genes exhibited consistent *cis*‐eQTL regulation, reflecting a spatially dispersed yet transcriptionally coordinated regulatory architecture within the pathway. The detection of *cis*‐eQTLs across multiple populations highlights the evolutionary conservation of this regulatory mechanism among wild and domesticated tomato genotypes (Figure 4c). However, transcript abundance varied substantially between populations, even for genes with conserved *cis*‐eQTLs, such as *Z‐ISO* (Figure 4d), suggesting population‐specific allelic effects that modulate carotenoid accumulation in different genetic backgrounds.

Among the wild tomato relatives analyzed, *S. pimpinellifolium* is notable for its high lycopene and β‐carotene content in red ripe fruits‒traits largely absent in green‐fruited species such as *S. pennellii*. *Z‐ISO* (*Solyc12g098710*), a key carotenoid biosynthesis gene, exhibited *cis*‐eQTLs in all populations except the Penne ILs. In contrast, *cis*‐regulation of *ZDS* (*Solyc01g097810*, *Solyc06g084610*), *CrtISO* (*Solyc10g081650*, *Solyc05g010180*, *Solyc02g085250*), and *LCYB* (*Solyc04g040190*) was mainly found in the Pimpi population (Figure 4c), suggesting that the accumulation of lycopene and β‐carotene in *S. pimpinellifolium* relies on *cis*‐regulation of genes across multiple steps of carotenogenesis. Within the Pimpi population, accessions carrying different alleles showed marked expression differences at these *cis*‐eQTLs (Figure S9). Analysis of carotenoid content data from a previous study [34] revealed significant variation in these metabolites between accessions with distinct *cis*‐eQTL alleles (Figure S10a). Moreover, strongly associated missense SNPs were identified within the *cis*‐eQTL regions of *ZDS* (*Solyc01g097810*) and *CrtISO‐L1* (*Solyc05g010180*), suggesting that these variants may simultaneously influence both gene expression and protein function, potentially contributing to natural variation in carotenoid accumulation and fruit color (Figure S11). In both Pimpi and Chees RILs, accessions with heterozygous genotypes exhibited intermediate expression of *Z‐ISO*, consistent with additive *cis*‐regulatory effects (Figure 4d). In contrast, no heterozygotes were detected in the Lycop ILs, likely reflecting the nature of introgression lines and suppressed recombination in those genomic regions. In the Pimpi population, accessions with the reference allele showed significantly reduced levels of phytoene and zeta‐carotene but significantly elevated lycopene content relative to the alternative allele (Figure 4e). Consistently, expression of *Z‐ISO* was strongly negatively correlated with phytoene and zeta‐carotene but positively correlated with lycopene (Figure S10b). These results further support *Z‐ISO* as the causal gene underlying *lyc12.1* [34], a major QTL previously shown to explain over 20% of lycopene variation in Pimpi RILs [35]. Together, these findings underscore a modular and evolutionarily stable regulatory framework governing carotenoid biosynthesis and provide valuable insights for dissecting and manipulating fruit color and nutritional quality traits in tomato.

### 2.6 *Cis*‐Regulation of Genes Controlling Fruit Sugars and Organic Acids

**FIGURE 5:**
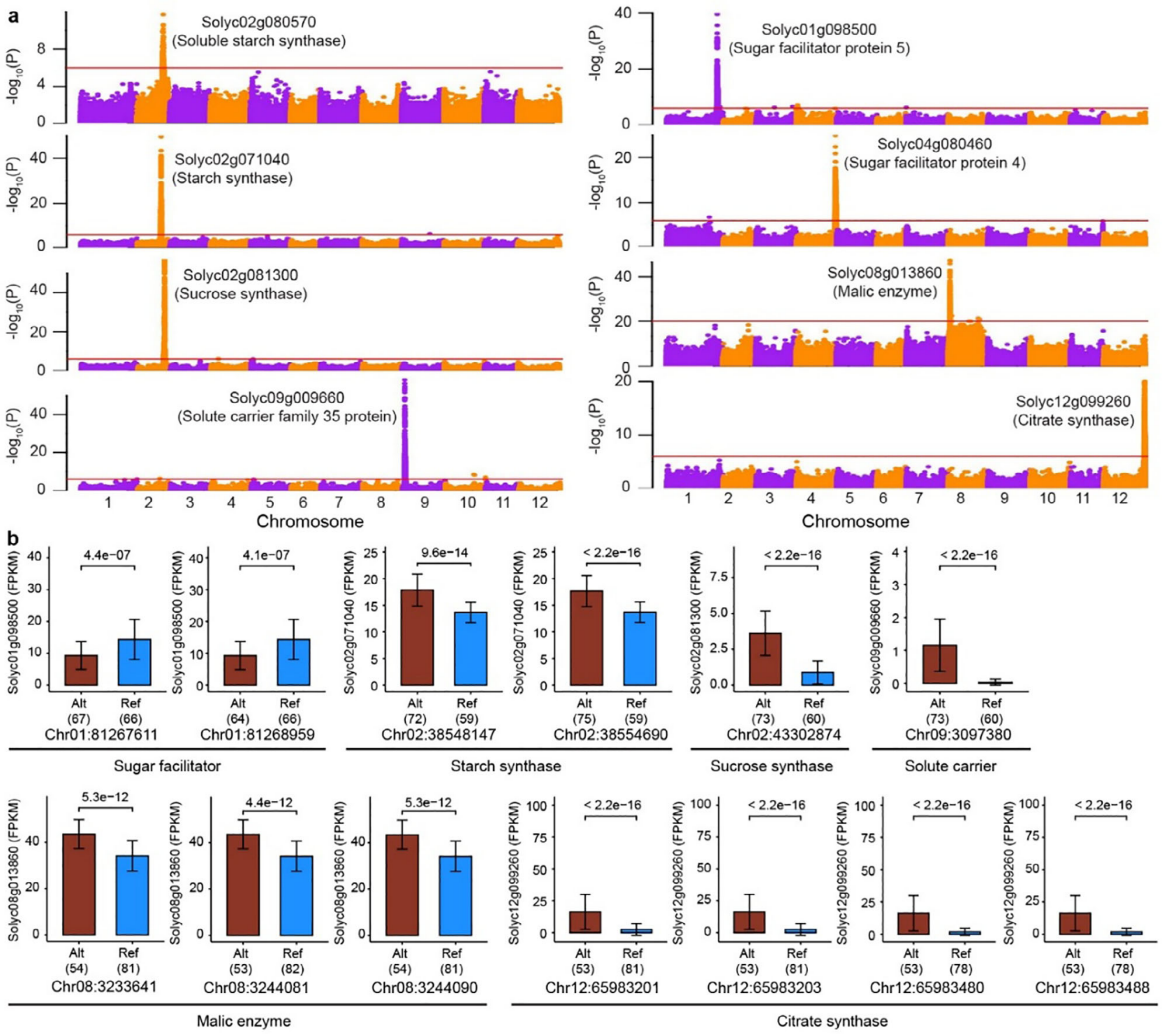
*Cis*‐regulatory variation of key genes associated with sugars and organic acids in tomato fruit. (a) Manhattan plots of eQTL meta‐analysis for genes involved in sugar and organic acid metabolism, highlighting strong *cis*‐eQTL signals. (b) Gene expression levels in accessions carrying reference (Ref) versus alternative (Alt) alleles at high‐effect SNP loci in the S. lycopersicum × *S. pimpinellifolium* RIL population. Data are presented as bar plots showing mean ± SD. *P*‐value were calculated using the Wilcoxon test. Sample sizes (n) are indicated in parentheses below each genotype.

Sugars and organic acids‒such as fructose, glucose, malate, and citrate‒not only define the nutritional quality of tomato fruit but also strongly influence consumer taste preferences [10, 36]. To elucidate the genetic basis of these traits, our eQTL meta‐analysis identified eight metabolism‐related genes involved in sugar and organic acid pathways that were associated with strong *cis*‐eQTLs (Figure 5a), suggesting that local regulatory variants are major contributors to the transcriptional variation of core metabolic genes in this trait class. Notably, the *cis*‐eQTL intervals of these genes harbored multiple putatively causal sequence variants, including missense mutations and high‐effect regulatory polymorphisms such as premature start codon gains within 5’ UTRs and splice region variants (Table S7). These variants were predominantly observed in the Pimpi RIL population and the natural diversity panel, likely reflecting a side effect of modern breeding, where prioritizing yield often came at the expense of fruit nutritional quality. In contrast, the high‐effect variants associated with sugar facilitator 4 that were specifically found in the Lycop population may represent valuable targets for improving the flavor and nutritional quality of modern cultivars. Analysis of metabolome profiling data of the natural population from a previous study [3] revealed that expression of the sugar facilitator gene *Solyc01g098500* was significantly negatively correlated with fructose and glucose levels, whereas expression of soluble starch synthase *Solyc02g080570* showed a significant positive correlation with these sugars (Figure S12). Significant expression differences were observed between accessions carrying different alleles of the peak SNPs in both the Pimpi (Figure 5b) and the natural population (Figure S13a). Notably, the presence of distinct haplotypes and diverse expression patterns associated with these variants in both the Pimpi (Figure S13b) and the natural population (Figure S13c) provides opportunities to fine‐tune sugar and organic acid contents for a balanced sugar/organic acid ratio to accommodate different consumer preferences. Collectively, these findings highlight the central role of *cis*‐regulatory polymorphisms in shaping the expression of sugar and acid metabolic genes and establish a genetic framework for marker‐assisted selection and allele‐specific genome editing to enhance tomato flavor and nutritional quality.

### 2.7 *Cis*‐ and *Trans*‐Regulatory Control of Gene Expression in the Folate Pathway

**FIGURE 6:**
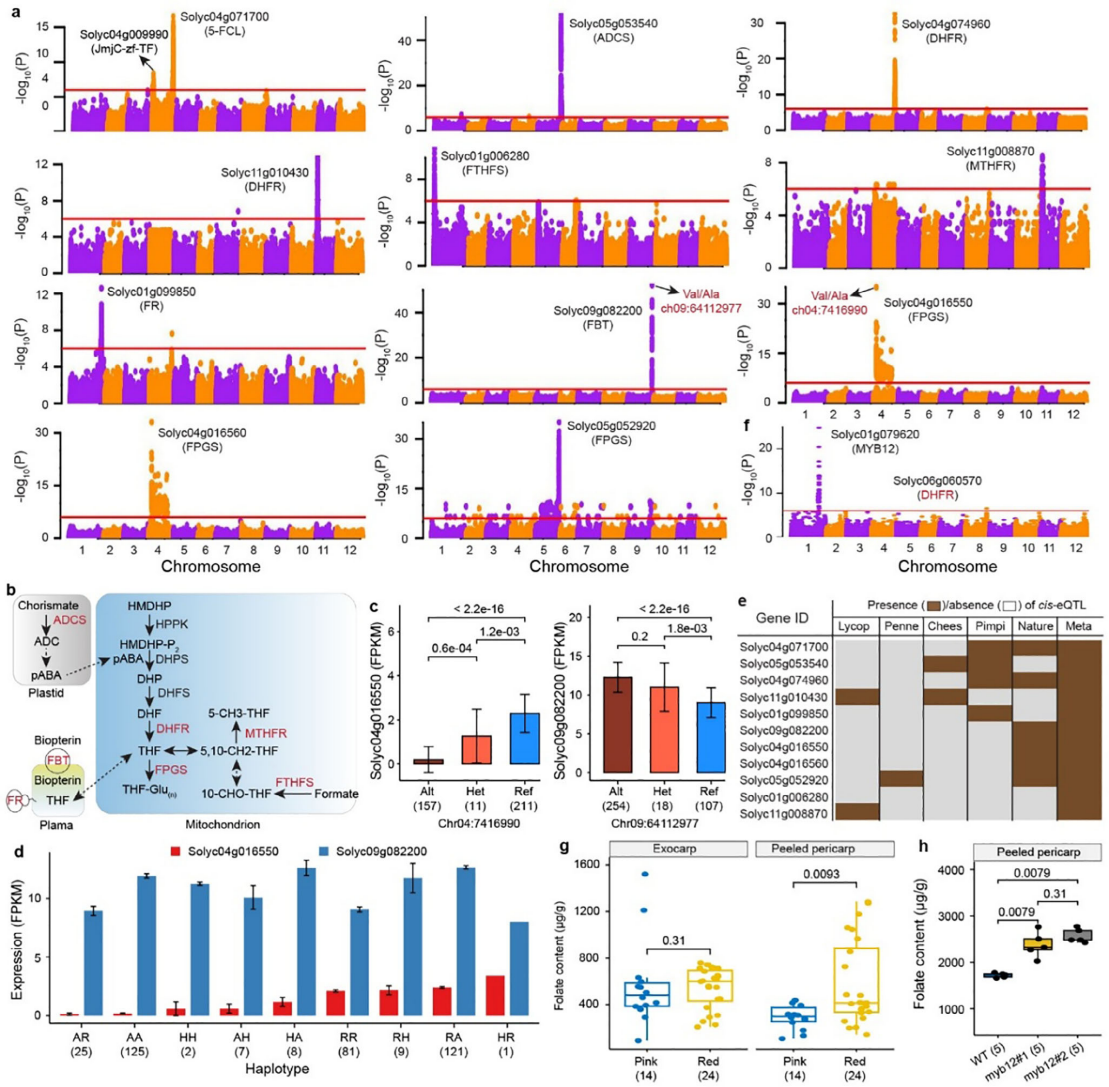
Genetic regulation of tomato folate metabolism revealed by eQTL meta‐analysis. (a) Manhattan plots of eQTLs meta‐analysis for key folate biosynthesis and transport genes, highlighting strong *cis*‐eQTL signals. Peak SNPs with missense mutations are highlighted in red. JmjC‐zf‐TF, jumonji/zinc finger transcription factor; 5‐FCL, 5‐formyltetrahydrofolate Cycloligase; ADCS, aminodeoxychorismate synthase; DHFR, dihydrofolate reductase; FTHFS, formate‐tetrahydrofolate ligase; MTHFR, methylenetetrahydrofolate reductase; FR, folate receptor; FBT, folate‐biopterin transporter; FPGS, folylpolyglutamate synthase. (b) Illustration of folate pathway in tomato, where genes with strong *cis*‐eQTL signals are shown in red. (c) Expression levels for *Solyc04g016550* (folylpolyglutamate synthase) and *Solyc09g082200* (folate‐biopterin transporter 1) in accessions carrying reference (Ref), alternative (Alt), or heterozygous (Het) alleles at the respective peak missense variants. Data are presented as bar plots showing mean ± SD. (d) Haplotype combinations of the two missense SNPs and their effects on expression of *Solyc04g016550* and *Solyc09g082200*. A, alternative allele; R, reference allele; H, heterozygous allele. (e) Population‐specific and shared *cis*‐ and *trans*‐eQTLs associated with folate‐related genes across different genetic backgrounds. (f) Manhattan plot of eQTL meta‐analysis for gene *Solyc06g060570* encoding dihydrofolate reductase. (g) Folate contents in the exocarp and peeled pericarp of pink and red tomatoes at the red‐ripe stage. (h) Folate contents in the peeled pericarp of Moneymaker wild‐type (WT) plants and two independent *MYB12* CRISPR/Cas9 mutant lines at the red‐ripe stage. For each boxplot in g and h, the lower and upper bounds indicate the first and third quartiles, respectively, the center line indicates the median, and the whiskers extend to 1.5× the interquartile range. *P*‐value were calculated using the Wilcoxon test. Sample sizes (n) are indicated in parentheses below or after each allele name (c) or genotype (g,h).

Folate (vitamin B9) is an essential metabolite with broad impacts on plant development and human health. Our eQTL meta‐analysis identified strong *cis*‐eQTLs for several genes involved in folate biosynthesis and metabolism (Figure 6a,b). These included genes encoding key biosynthetic enzymes, such as 5‐formyltetrahydrofolate cycloligase (*Solyc04g071700*), aminodeoxychorismate synthase (*Solyc05g053540*), dihydrofolate reductase (*Solyc04g074960*, *Solyc11g010430*), and folylpolyglutamate synthases (*Solyc04g016550*, *Solyc04g016560*, *Solyc05g052920*). In addition, we identified *cis*‐eQTLs for genes involved in folate transport and binding, including a folate receptor (*Solyc01g099850*) and folate‐biopterin transporter 1 (*Solyc09g082200*). Among these, two *cis*‐eQTLs‒targeting *Solyc04g016550* (folylpolyglutamate synthase) and *Solyc09g082200* (folate‐biopterin transporter 1)‒were driven by high‐effect missense SNPs, both resulting in valine‐to‐alanine substitutions (Figure 6a). Interestingly, these variants exhibited opposing effects on gene expression: accessions carrying the alternative alleles showed significantly lower expression of *Solyc04g016550* and significantly higher expression of *Solyc09g082200*, compared to those with the reference alleles (Figure 6c). Haplotype analysis of these two loci revealed diverse allelic combinations associated with substantial differences in transcript abundance, offering natural variation that could be leveraged to optimize folate biosynthesis (Figure 6d). These *cis*‐eQTLs were not limited to individual populations; several were consistently detected across multiple genetic backgrounds, underscoring their evolutionary conservation and regulatory stability (Figure 6e).

Besides local regulatory effects, we also identified a strong *trans*‐eQTL associated with the expression of *Solyc06g060570*, which encodes dihydrofolate reductase (Figure 6f), a key rate‐limiting enzyme in the folate biosynthetic pathway (Figure 6b). Interestingly, this *trans*‐eQTL region harbored *Solyc01g079620* (*MYB12*), a transcription factor previously characterized for its role in flavonoid biosynthesis [3, 19, 20] (Figure 3). Supporting this regulatory relationship, a prior study [3] showed that *Solyc06g060570* expression was abolished in MYB12 mutant lines (Table S6). Additionally, *Solyc05g053540*, which encodes aminodeoxychorismate synthase, also showed markedly reduced expression in the same mutant background [3]. Furthermore, *MYB117* (*Solyc05g007870*), another MYB transcription factor, has been shown to increase folate content in tomato [37]. Although *MYB117* was not identified as a direct *trans*‐regulated target of *MYB12*, another MYB, *Solyc06g065100*, was identified as the direct *trans*‐regulated target (Figure 3) and belonged to the *MYB12* co‐expression module, suggesting that multiple MYB family members may contribute to the transcriptional regulation of folate metabolism in tomato. In addition, the *trans*‐eQTL associated with the expression of *Solyc04g071700* (encoding 5‐formyltetrahydrofolate cycloligase) harbored *Solyc04g009990*, which encodes a Jumonji/zinc finger transcription factor known to play important roles in chromatin regulation and development [38]. The significant negative correlation between *Solyc04g071700* and *Solyc04g009990* aligns with the established function of Jumonji/zinc finger proteins as transcriptional repressors (Figure S14), suggesting that chromatin‐modifying Jumonji TFs may directly regulate folate biosynthetic genes.

Because *MYB12* is the causal gene underlying pink fruits and also potentially functions as a *trans*‐regulator of folate biosynthesis, we hypothesized that folate levels would differ between pink and red tomatoes. To test this, we quantified folate content in both the exocarp and peeled pericarp tissues of pink and red tomato cultivars. We found that while folate levels in exocarp did not differ significantly, pink tomatoes exhibited a significantly lower folate content in peeled pericarp (Figure 6g). To further validate the function of *MYB12* in regulating folate metabolism, we measured folate content in the peeled pericarp of Moneymaker wild‐type (WT) plants and two independent *MYB12* CRISPR/Cas9 mutant lines generated in our previous study [3]. Both mutants exhibited a significant increase in folate levels, with an average increase of approximately 43.5% compared to WT (Figure 6h). Together, these findings reveal a nuanced yet evolutionarily conserved regulatory architecture underlying folate accumulation in tomato, involving both *cis*‐acting variants at core biosynthetic genes and long‐range *trans*‐regulatory mechanisms potentially mediated by pleiotropic MYB transcription factors.

## 3 Discussion

This study presents the first eQTL meta‐analysis framework spanning multiple populations to date in tomato, providing unprecedented genetic insights into the transcriptional regulatory architecture underlying fruit development and nutritional quality. By integrating five genetically and evolutionarily diverse populations‒including interspecific introgression lines (ILs), recombinant inbred lines (RILs), and a natural diversity panel‒we identified more than 40 000 eQTLs, comprising 9463 *cis‐*eQTLs and 31 465 *trans‐*eQTLs. Notably, a substantial number of eQTLs were uniquely discovered through meta‐analysis, highlighting its power to uncover both conserved and population‐specific regulatory variants that are often missed in single‐population studies [9, 10].

Our analysis reveals a dynamic and complex regulatory landscape of TFs, shaped by evolutionary divergence and domestication. Among the 881 TFs associated with eQTLs, families such as MYB, C2H2, bHLH, and NAC were prominently represented, underscoring their central roles in fruit development, hormone signaling, and chromatin remodeling [29, 30, 31]. The presence of both conserved cis‐eQTLs shared across populations and population‐specific TF‐associated eQTLs reflects regulatory and adaptive flexibility during tomato evolution, exemplified by loci such as EIL3 [29], ej2 [30], and FUL2/MAP7 [31]. Notably, we identified a *trans*‐eQTL hotspot regulating a cluster of TFs and harboring a gene encoding ent‐kaurene synthase‒a key GA biosynthetic enzyme‒suggesting integration of hormone signaling with chromatin remodeling and stress‐response networks during tomato fruit ripening [32, 33].

Beyond TF regulation, our eQTL meta‐analysis reveals key *cis*‐regulatory mechanisms controlling major fruit nutritional quality traits. Genes involved in carotenoid, sugar, organic acid, and folate metabolism were predominantly regulated by strong *cis*‐eQTLs. A number of these regulatory variants were shared across populations, demonstrating the power of eQTL meta‐analysis and shedding light on the evolutionary dynamics of transcriptional regulation in these pathways [3, 14, 16, 17]. Importantly, we identified high‐effect *cis*‐regulatory variants within 5’ UTRs and coding regions, including missense mutations and premature start codon gain variants, suggesting possible direct transcriptional and functional consequences. These alleles offer promising targets for precision breeding and genome editing aimed at improving fruit nutritional quality and tailoring traits to meet diverse consumer preferences [10, 17, 36].

Our findings also highlight the regulatory impact of *trans*‐eQTLs on complex metabolic pathways. Among these, *MYB12* emerged as a key *trans*‐regulatory hub. While previously known for its role in regulating flavonoid metabolism [3, 18, 19, 20], our study expands its regulatory scope to include genes involved in upstream precursors of flavonoids and folate biosynthesis. GO and KEGG enrichment of *MYB12* co‐expression module suggests that this TF may coordinate broad metabolic reprogramming beyond specialized pathways such as flavonoid and folate metabolism. Notably, the *MYB12*‐associated *trans*‐eQTL hotspot also co‐localized with multiple co‐expressed regulatory genes, including additional MYBs, F‐box genes, GRAS‐domain TFs, heat shock TFs, and hormone signaling‐related genes. This hotspot likely orchestrates broad transcriptional reprogramming through (1) direct regulation of metabolic pathway genes [3, 21], (2) coordinated regulation with other TFs [39, 40], and (3) crosstalk with hormone signaling pathways [41]. GRAS transcription factors have been reported to play multifaceted roles in plant growth, development, and resistance to various biotic and abiotic stresses [42, 43], while heat shock TFs play a crucial role in plant response to abiotic stresses [44]. The coordinated expression of MYBs, F‐box genes, GRAS‐domain TFs, and hormone regulators supports a multifaceted regulatory module that may balance the trade‐off between tomato nutritional quality (e.g., folate and flavonoid) and stress tolerance. The eQTLs identified here generate specific, testable hypotheses regarding the regulation of key metabolic and developmental pathways. Future work, including targeted metabolomic profiling and functional validation of candidate regulators, will be essential to fully elucidate the molecular mechanisms underlying these associations. Furthermore, the characterization of *MYB12* CRISPR/Cas9 mutant lines and natural populations with pink‐ and red‐colored fruits provides strong evidence supporting the role of MYB12 in regulating flavonoid and folate biosynthesis; however, further validation across additional genetic backgrounds would strengthen this conclusion.

In conclusion, our eQTL meta‐analysis provides a high‐resolution atlas of gene expression regulation in tomato, capturing both conserved and population‐specific regulatory variation. The discovery of major *cis‐* and *trans*‐eQTLs associated with TFs, hormone signaling, and key metabolic pathways‒including those for carotenoids, flavonoids, sugars, organic acids, and folate‒offers valuable insights into the genetic architecture underlying fruit nutritional quality. This work establishes a foundational resource for dissecting transcriptional networks and accelerates the path toward targeted crop improvement through molecular breeding and precision genome editing.

## 4 Materials and Methods

### 4.1 Plant Growth, RNA Isolation and Sequencing

Five tomato populations were used in this study: two interspecific introgression line (IL) populations derived from crosses of *S. lycopersicum* × *S. lycopersicoides* LA2951 (Lycop; n = 106) and *S. lycopersicum* × *S. pennellii* LA0716 (Penne; n = 76); two recombinant inbred line (RIL) populations from crosses of *S. lycopersicum* × *S. pimpinellifolium* LA2093 (Pimpi; n = 136) and *S. lycopersicum* × *S. cheesmaniae* LA483 (Chees; n = 59); and a natural diversity panel consisting of 379 *S. pimpinellifolium*, *S. lycopersicum* var. *cerasiforme*, and *S. lycopersicum* accessions (Table S2). Plants from the two IL and two RIL populations were grown in open fields. Fruits at the ripe stage were harvested, flash‐frozen in liquid nitrogen, and stored at −80°C before use. Total RNA was extracted from fruit pericarp using the RNeasy Plant Mini Kit (QIAGEN). Strand‐specific RNA‐Seq libraries were constructed following the protocol described in Zhong et al. [45] and sequenced on the Illumina NextSeq 500 platform. RNA‐Seq data from RILs of *S. lycopersicum* × *S. pimpinellifolium* were also used for QTL mapping of volatiles and carotenoids in our earlier study [46]. For the natural diversity panel, RNA‐Seq data from orange‐stage fruits were obtained from a previous study [3] (NCBI accession PRJNA396272). Additionally, RNA‐Seq data were generated for fruit at the mature green stage of the *S. lycopersicum* × *S. cheesmaniae* RIL population, which were used exclusively in SNP calling (Table S2).

### 4.2 RNA‐Seq Read Mapping and SNP Calling

Raw Illumina reads were first processed to remove adaptors and low‐quality sequences using Trimmomatic [47] (v0.39) with parameters ‘TruSeq3‐PE‐2.fa:2:30:10:1:TRUE SLIDINGWINDOW:4:20 LEADING:3 TRAILING:3 MINLEN:40’. The cleaned reads were then mapped to the tomato ‘Heinz 1706’ reference genome [48] (v4.0) using STAR [49] with two‐pass mode (v2.7.0a). Finally, alignment of BAM files from biological replicates or different tissues were merged into a single BAM file for each accession for downstream analyses. Raw read counts for each gene were calculated using StringTie [50] (v2.2.3) and then normalized to fragments per kilobase of transcript per million mapped fragments (FPKM). For each population, a GVCF file was generated for each accession using the ‘HaplotypeCaller’ function implemented in GATK [51] (v3.8) with parameters ‘–genotyping_mode DISCOVERY –max_alternate_alleles 3 –read_filter OverclippedRead’. SNPs across each population were then called using the function ‘GenotypeGVCFs’ with default parameters. Hard filtering was applied to the resulting raw SNP set using GATK with parameters ‘QD < 2.0 | | FS > 60.0 | | MQ < 40.0 | | MQRankSum < −12.5 | | ReadPosRankSum < −8.0’.

### 4.3 Imputation of Missing Genotypic Data

To improve SNP genotyping quality, missing genotypes were imputed for each population using Beagle [52] (v5.3) with default settings. To evaluate imputation accuracy, 10%, 20%, and 30% of SNP sites were randomly masked as missing genotypes and subsequently imputed. For downstream analyses, only biallelic SNPs with a minor allele frequency ≥ 1% in the nature population and a minor allele count ≥1 in the RIL and IL populations were retained. SNPs were functionally annotated using SnpEff [53] (v5.0e) with default parameters. SNPs predicted to have missense, splicing site, or start/stop gain/loss effects were considered as high‐effect candidates.

### 4.4 eQTL Analysis

eQTL mapping for each expressed gene was performed using EMMAX [54] (v20120210). To account for hidden and confounding factors, normal quantile‐transformed expression values were processed using the probabilistic estimation of expression residuals (PEER) method [55], and the top 20 factors were included as covariates in the association model. In addition, the first five principal components (PCs), calculated using PLINK [56] with the parameter ‘–pca’, along with the Balding‐Nichols kinship matrix constructed using EMMAX [54] with parameters ‘‐v ‐d 10’, were also included as cofactors in the model to account for population structure.

### 4.5 eQTL Meta‐Analysis

To perform eQTL meta‐analysis, the genomic inflation factor (λ) was estimated for each individual eQTL study and used to adjust the standard errors (SE) of the beta coefficients using the formula SE × $\sqrt{\lambda }$. The meta‐analysis was performed following a previously established pipeline [10]. Briefly, meta‐analysis was first performed using the inverse variance‐weighted fixed‐effect model implemented in METAL [57]. For SNPs with different allele combinations across populations, meta‐analysis was performed separately, and only the most significant result was retained for downstream analyses. For SNPs showing heterogeneity (I^2^ > 25, indicating moderate heterogeneity), the Han and Eskin random‐effect model implemented in METASOFT [58] was used. Results from METAL with I^2^ ≤ 25 and from METASOFT with I^2^ > 25 were combined to generate the final set of meta‐analysis results. Significance thresholds were determined using the Bonferroni correction, with α  =  0.05 for significant associations and α  =  1 for suggestive associations. SNPs in close physical proximity (50 kb) and associated with the expression of the same gene were grouped into an eQTL block, represented by the most significant SNP (lead SNP) in the block. eQTLs located within 50 kb of transcription start sites or transcription stop sites of genes were classified as *cis‐*eQTLs, while those located ≥ 50 kb away or on different chromosomes were designated as *trans‐*eQTLs.

### 4.6 Weighted Gene Co‐Expression Network Analysis

Weighted gene co‐expression networks were constructed for each population using the WGCNA R package [59] (v1.72‐1). The pickSoftThreshold function was used to determine the optimal soft‐thresholding power (β = 10), at which the scale‐free topology fit index (R^2^) exceeded 0.85 with negligible improvement in mean connectivity (module size) [38]. Expression modules were defined with a minimum merged module size of 30 and a dissimilarity cutoff of 0.25. GO and KEGG enrichment analysis was performed using the clusterProfiler R package (v4.18.4) [60].

### 4.7 Folate Measurement

Folate content was measured in both exocarp and peeled pericarp at the ripe stage of pink (n = 7) and red (n = 12) cultivated tomatoes, as well as Moneymaker and two *MYB12* CRISPR/Cas9 mutant lines generated in our previous study [3]. After being frozen in liquid nitrogen, the tissues were homogenized using a Supor blender, transferred to 50 mL centrifuge tubes, and stored at −24°C till use. For folate extraction, 0.1 g of homogenized sample was placed in a 10 mL centrifuge tube and extracted with 5 mL of ammonia solution (prepared by mixing 4900 µL ultrapure water with 100 µL concentrated ammonium hydroxide). Samples were sonicated in a KQ‐500DE digital ultrasonic bath for 10 min, with gentle agitation applied twice during the extraction. The extracts were then centrifuged at 4000 rpm for 5 min. The resulting supernatant was then transferred to amber autosampler vials for measurement. Fluorescence intensity was recorded using a TECAN Infinite M200 Pro microplate reader at excitation/emission wavelengths of 370/455 nm. A standard curve was generated using serial dilutions of folate (0.1, 1.0, 10, and 100 µmol/L) in ammonia solution, with fluorescence intensity plotted against known concentrations to quantify sample folate levels. All measurements were performed in three technical replicates.

### 4.8 Statistical Analysis

Data were analyzed using R software (version 4.5.2) with the dplyr (version 1.1.4), ggpubr (version 0.6.2) and ggplot2 (version 4.0.1) packages, as well as Microsoft Excel and other specialized software as noted above. Data distributions were assessed for normality, and non‐normally distributed data (e.g., gene expression data) were transformed (e.g., using normal quantile transformation) prior to parametric testing. Data in bar plots are presented as mean ± standard deviation (SD), with specific sample sizes (n) for each experiment provided in figure legends or method descriptions. Boxplots display the 25th, 50th, and 75th percentiles, with jittered points representing individual data points. For statistical analysis, the Wilcoxon test was used to compare groups, and Bonferroni correction was applied to adjust *P*‐values for multiple comparisons and control the false positive rate. A *P*‐value < 0.05 was considered statistically significant. Gene ontology and pathway enrichment analyses were performed using a hypergeometric test, with a false discovery rate (FDR) < 0.05 considered significant.

## Author Contributions

Z.F., J.J.G., and S.H. designed and managed the project. R.M., J.L., I.G., AF., Y.X., D.M.T., S.H., J.J.G., and H.K. collected samples, constructed RNA‐Seq libraries, and coordinated sequencing. J.Zhao, B.W., Q.Y., and J.Zhang performed mutant characterization and folate measurement. X.W. and J.Zhao performed data analyses. J.Z. and X.W. wrote the manuscript. Z.F. revised the manuscript.

## Conflicts of Interest

The authors declare no conflicts of interest.

## Supporting information




**Supporting File 1**: advs75689‐sup‐0001‐SuppMat.docx.

**Supporting File 2**: advs75689‐sup‐0002‐TableS1‐S7.xlsx.

## Data Availability

Raw RNA‐Seq reads have been deposited in the National Center for Biotechnology Information BioProject database under accessions nos. PRJNA1314267, PRJNA526494, and PRJNA509236.
