# Supplementary material for: eQTL Meta‐Analysis Reveals Conserved and Population‐Specific Regulatory Variation Underlying Nutritional Trait Evolution and Domestication in Tomato
Source: Adv Sci (Weinh). 2026 May 14:e19899. Online ahead of print. doi: 10.1002/advs.202519899 (PMC13335988; doi:10.1002/advs.202519899)
Supplement: Supplementary file 1 — Supporting File 1: advs75689‐sup‐0001‐SuppMat.docx. [file ADVS-9999-e19899-s002.docx]

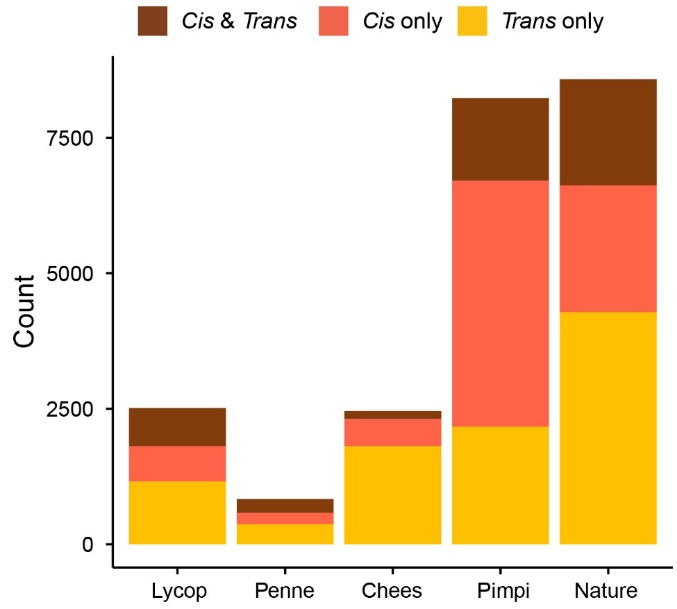


**Supplementary Fig. 1** Number of genes associated with *cis-*eQTLs only, *trans-*eQTLs only, or both *cis-* and *trans-*eQTLs.


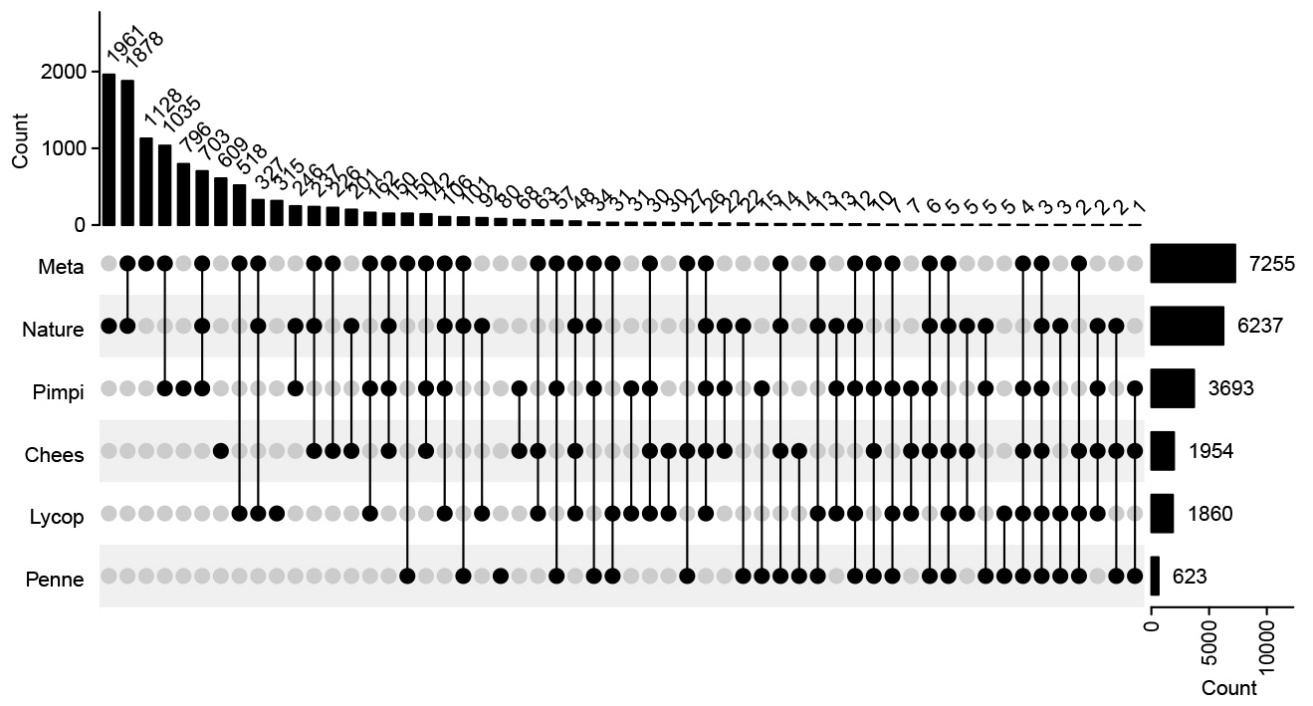


**Supplementary Fig. 2** UpSet plot of genes associated with *trans*-eQTLs.


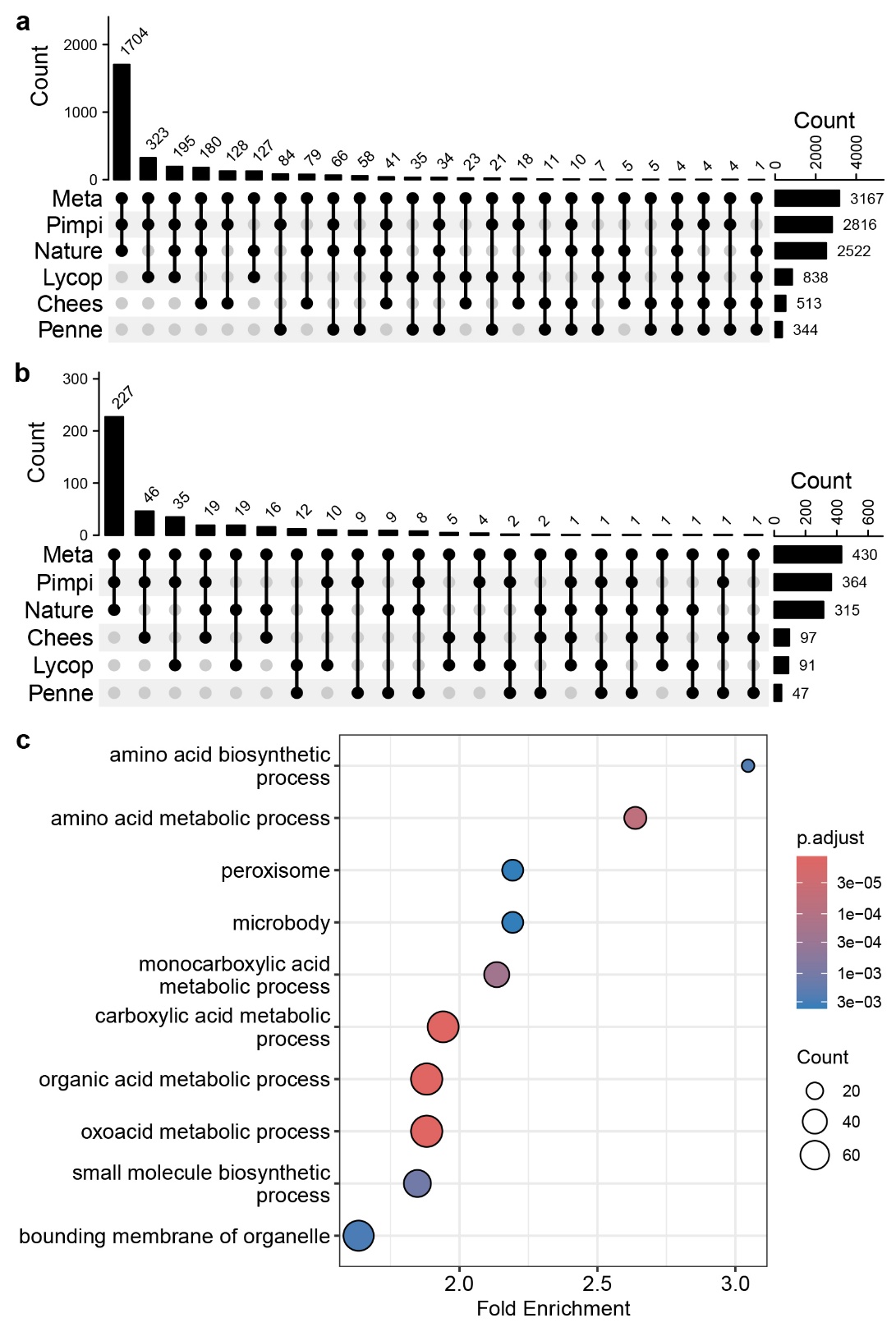


**Supplementary Fig. 3** Distribution and GO enrichment analysis of genes associated with conserved eQTLs. **a**, UpSet plot of genes associated with conserved *cis*-eQTLs; **b**, UpSet plot of genes associated with conserved *trans*-eQTLs; **c**, GO terms enriched in genes associated with conserved *cis*-eQTLs.


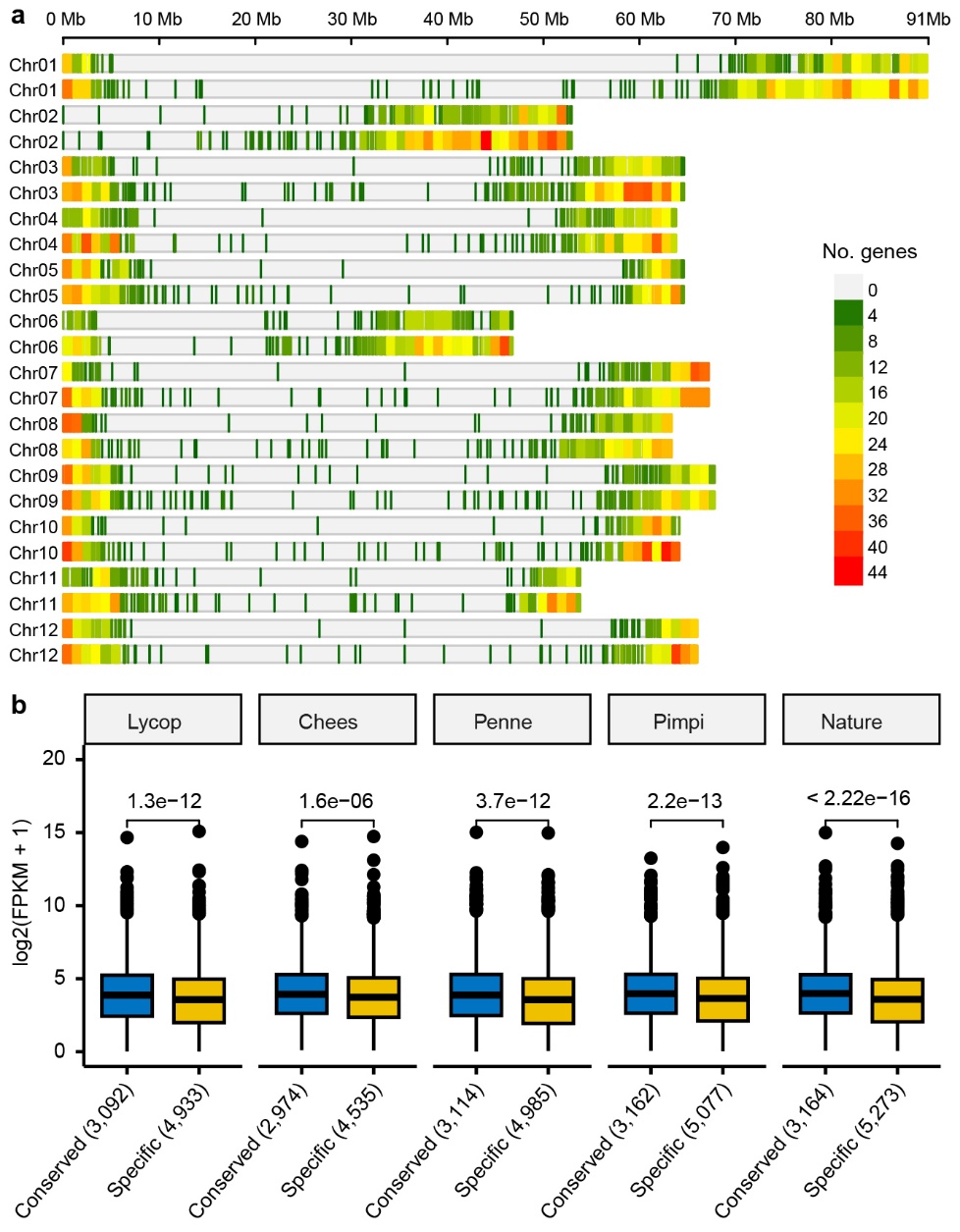


**Supplementary Fig. 4** Genomic distribution and expression characteristics of genes associated with conserved versus population-specific *cis*-eQTLs. **a**, Genome-wide density distribution of genes associated with conserved and population-specific *cis*-eQTLs (gene density = number of genes per 1-Mb window). For each chromosome (Chr01–Chr12), the upper panel shows the density of genes associated with conserved *cis*-eQTLs, and the lower panel shows the density of genes associated with population-specific *cis*-eQTLs. **b**, Comparison of expression levels of genes associated with conserved versus population-specific *cis*-eQTLs across different populations. For each boxplot, the lower and upper bounds indicate the first and third quartiles, respectively, the center line indicates the median, and the whiskers extend to 1.5× the interquartile range. *P*-values were calculated using the Wilcoxon test. Sample sizes (n) are indicated in parentheses after *cis*-eQTL types (conserved/specific).


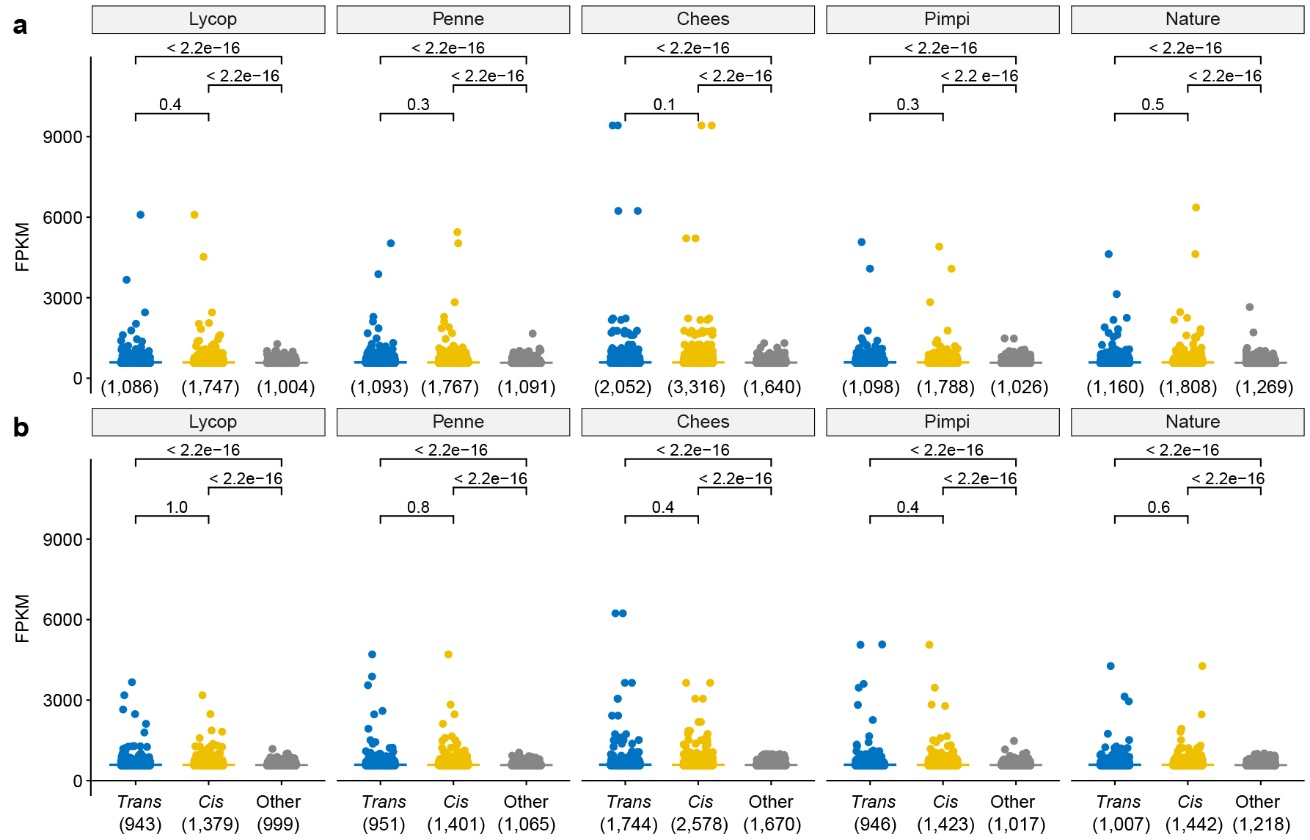


**Supplementary Fig. 5** Comparison of expression levels of genes associated with *cis*-eQTLs (*Cis*), *trans*-eQTLs (*Trans*), and genes without eQTLs (Other) within domesticated (**a**) and improvement sweeps (**b**) across different populations. For each boxplot, the lower and upper bounds indicate the first and third quartiles, respectively, the center line indicates the median, and the whiskers extend to 1.5× the interquartile range. *P*-values were calculated using the Wilcoxon test. Sample sizes (n) are indicated in parentheses.


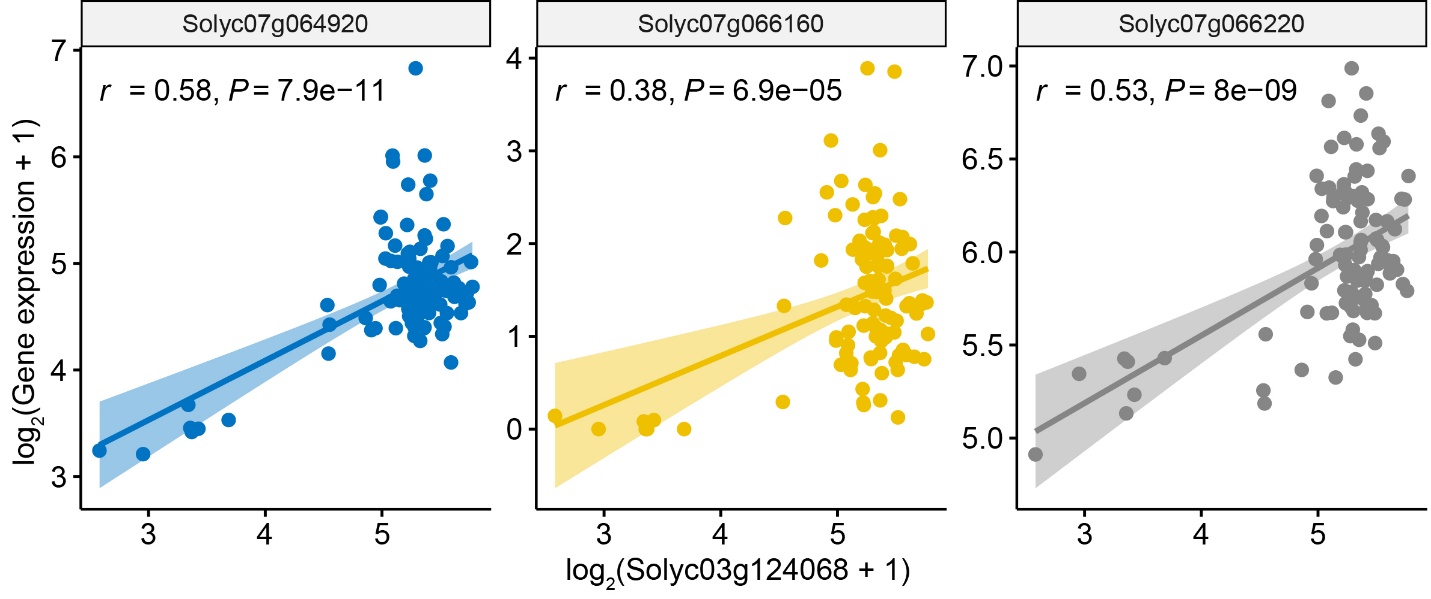


**Supplementary Fig. 6** Expression correlation between *Solyc03g124068* and *Solyc07g064920*, *Solyc07g066160* and *Solyc07g066220*.


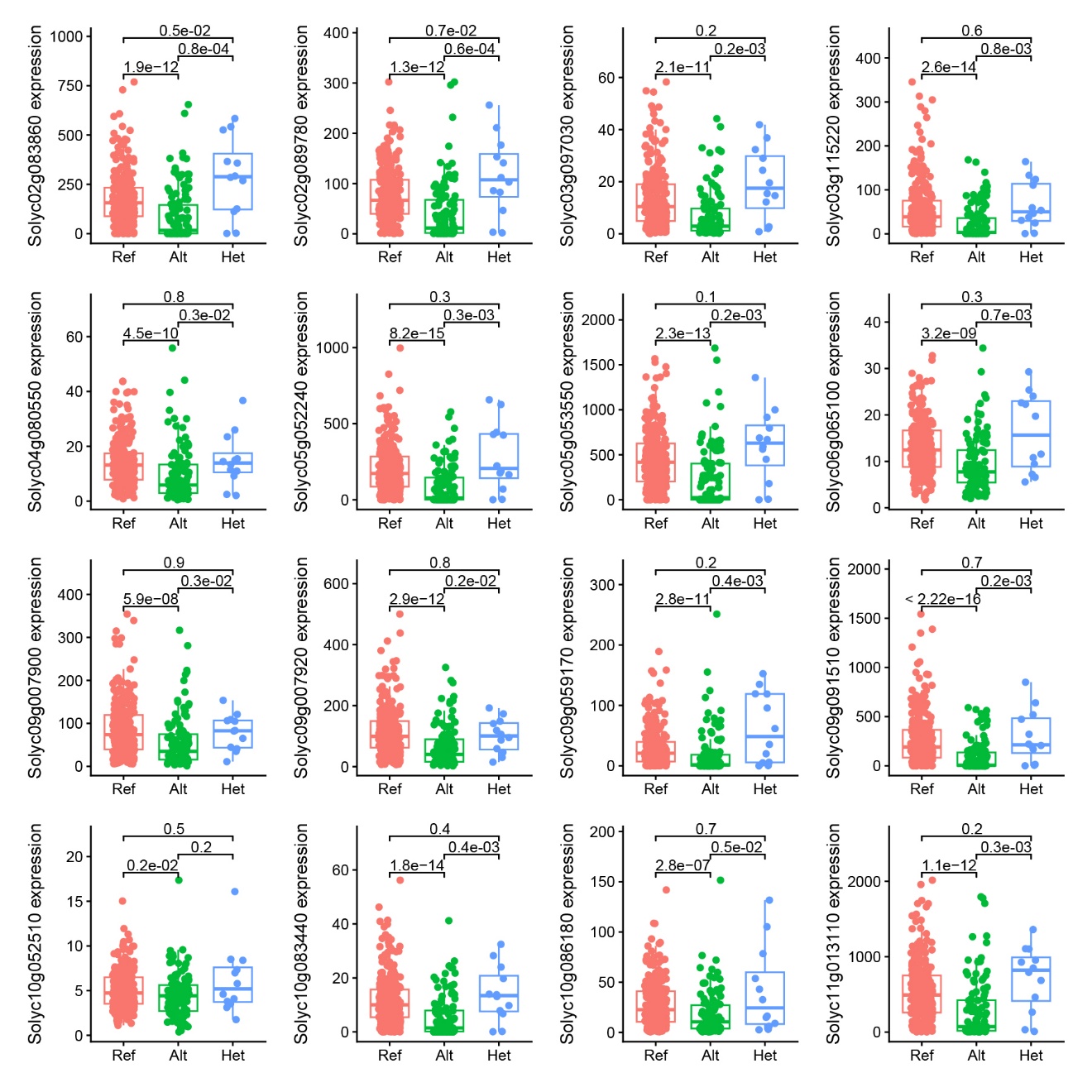


**Supplementary Fig. 7** Expression differences of target genes in the flavonoid biosynthetic pathway between accessions carrying the reference allele (Ref, n = 255), alternative allele (Alt, n = 112), and heterozygous allele (Het, n = 12) for the peak SNPs associated with *MYB12* in the natural population. For each boxplot, the lower and upper bounds indicate the first and third quartiles, respectively, the center line indicates the median, and the whiskers extend to 1.5× the interquartile range. *P*-values were calculated using the Wilcoxon test.


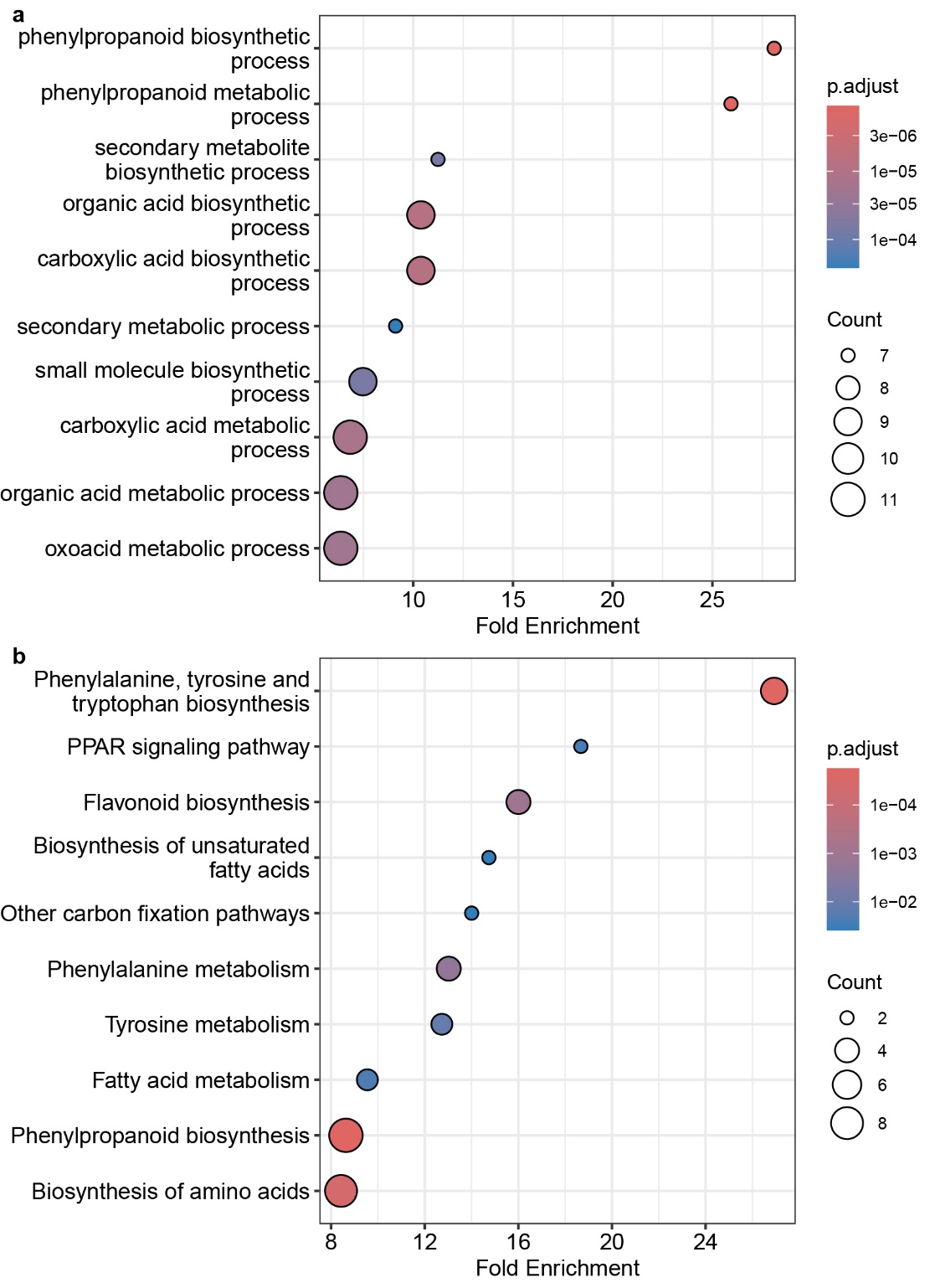


**Supplementary Fig. 8** GO and KEGG enrichment analyses for genes within the *MYB12* co-expression module. **a**,**b** GO terms (**a**) and KEGG pathways (**b**) enriched in genes of the MBY12 co-expression module.

**
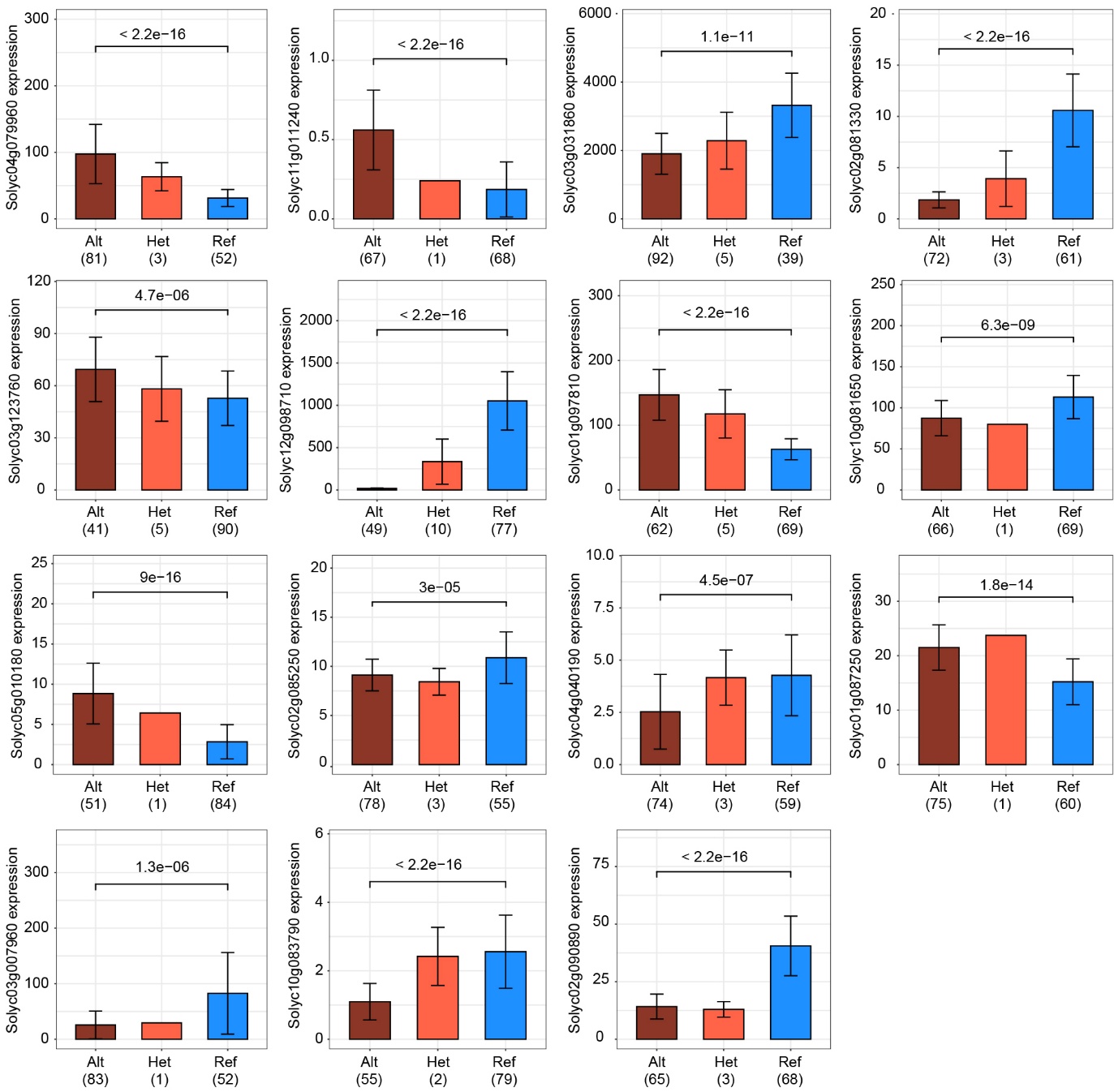
**

**Supplementary Fig. 9** Expression levels of carotenoid biosynthetic genes in Pimpi accessions carrying the reference (Ref), heterozygous (Het), and alternative (Alt) alleles at the most significant high-effect missense SNP loci of the corresponding *cis*-eQTLs. Data are presented in bar plots with mean ± SD. *P*-values were calculated using the Wilcoxon test. Sample sizes (n) are indicated in parentheses below each genotype.


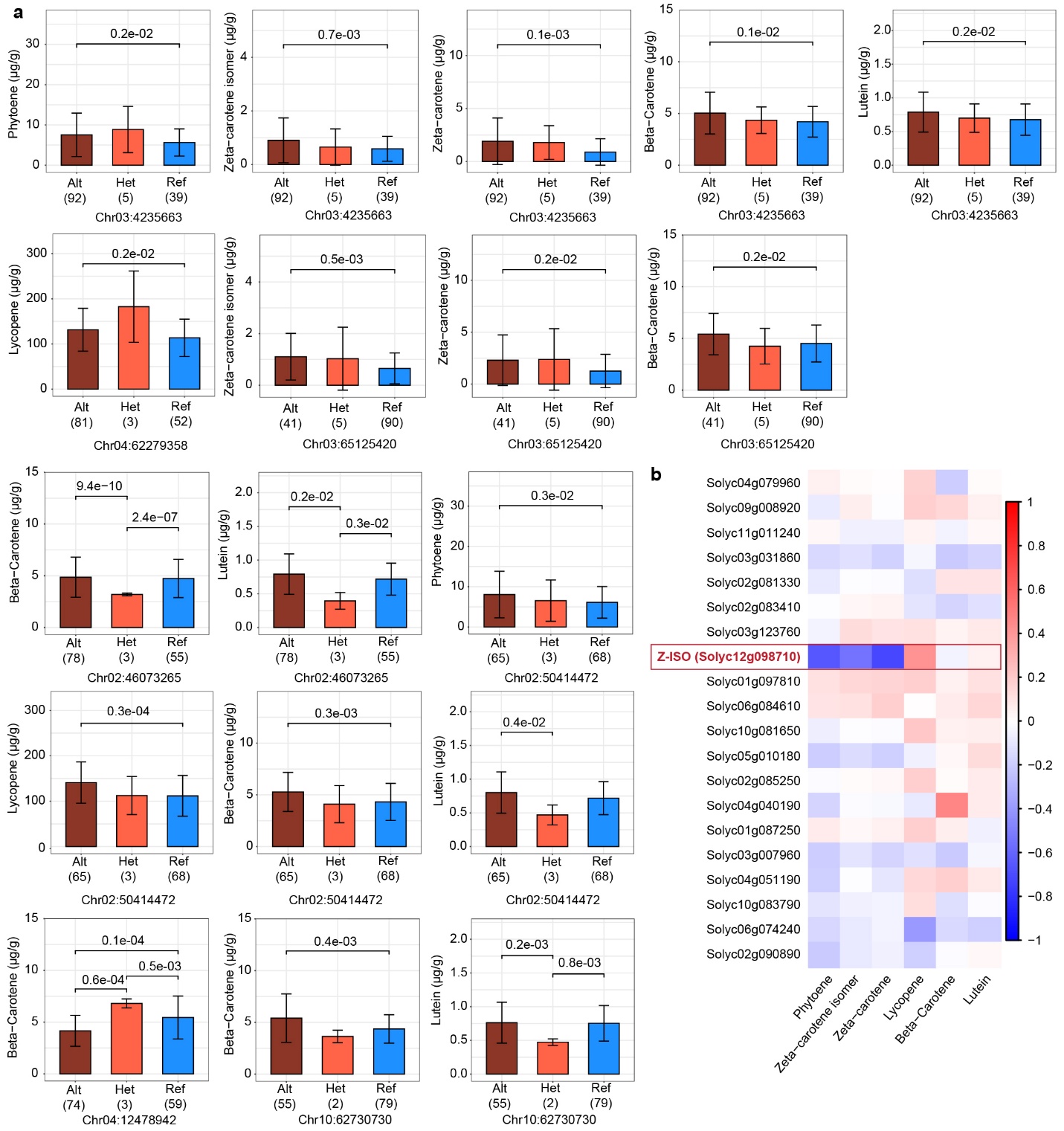


**Supplementary Fig. 10** Carotenoid variation and correlations with genes in the carotenoid pathway associated with *cis*-eQTLs. **a**, Carotenoid contents in Pimpi RIL accessions carrying reference (Ref), heterozygous (Het), or alternative (Alt) alleles at the most significant SNP loci. Only statistically significant differences are indicated in the plots. **b**, Correlations between carotenoid contents and expression of genes associated with *cis*-eQTLs. Data are presented in bar plot with mean ± SD. *P*-values were calculated using the Wilcoxon test. Sample sizes (n) are indicated in parentheses below each genotype.


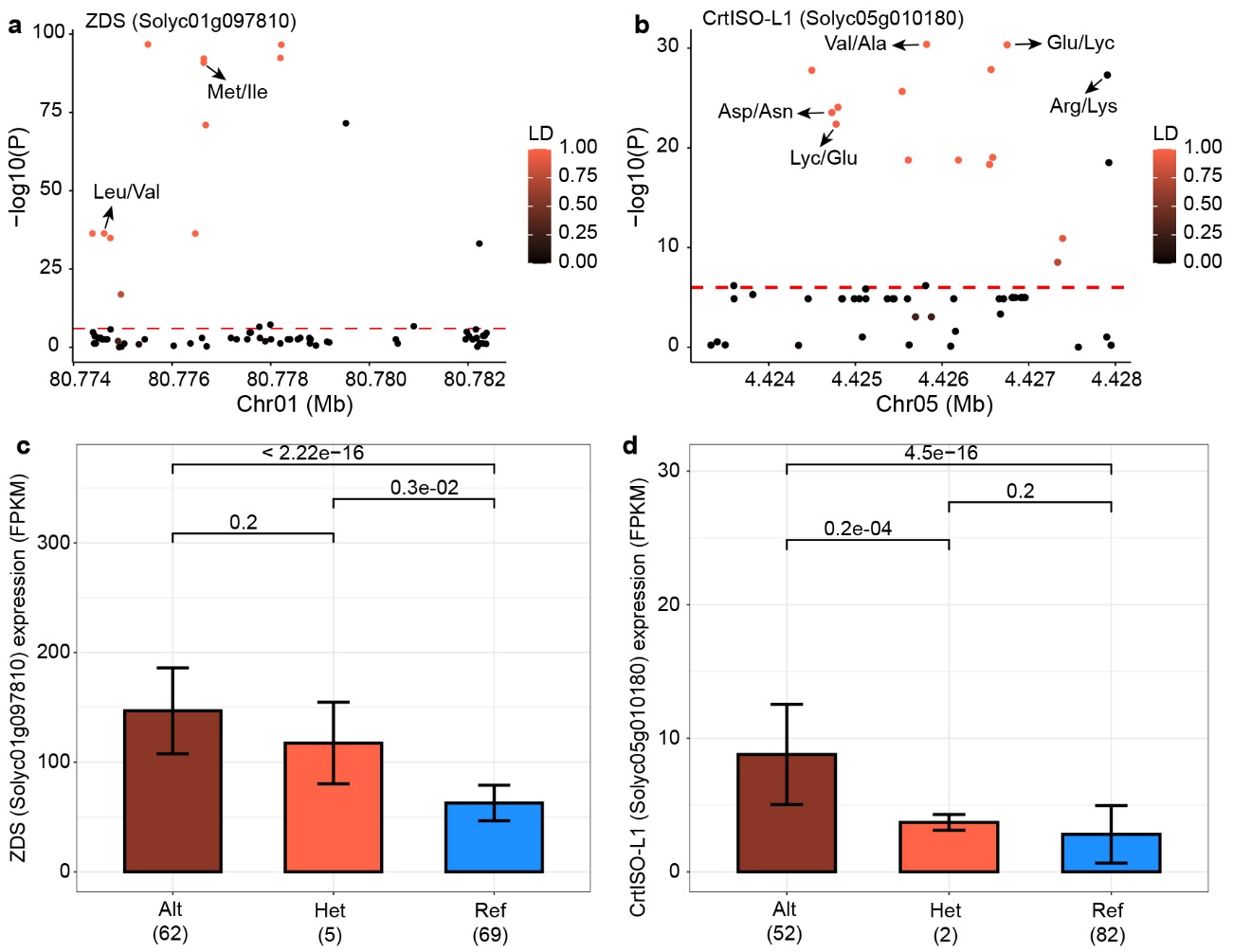


**Supplementary Fig. 11** eQTLs for *ZDS* (*Solyc01g097810*) and *CrtISO-L1* (*Solyc05g010180*). **a**,**b**, Regional Manhattan plots of meta-eQTL analysis for *ZDS* (**a**) and *CrtISO-L1* (**b**). Black arrows indicate amino acid changes caused by the mutations. **c**,**d**, Expression levels of *ZDS* (**c**) and *CrtISO-L1* (**d**) in accessions carrying reference (Ref), heterozygous (Het), or alternative (Alt) alleles at the most significant high-effect missense SNP loci. Data are presented in bar plot with mean ± SD. *P*-values were calculated using the Wilcoxon test. Sample sizes (n) are indicated in parentheses below each genotype.


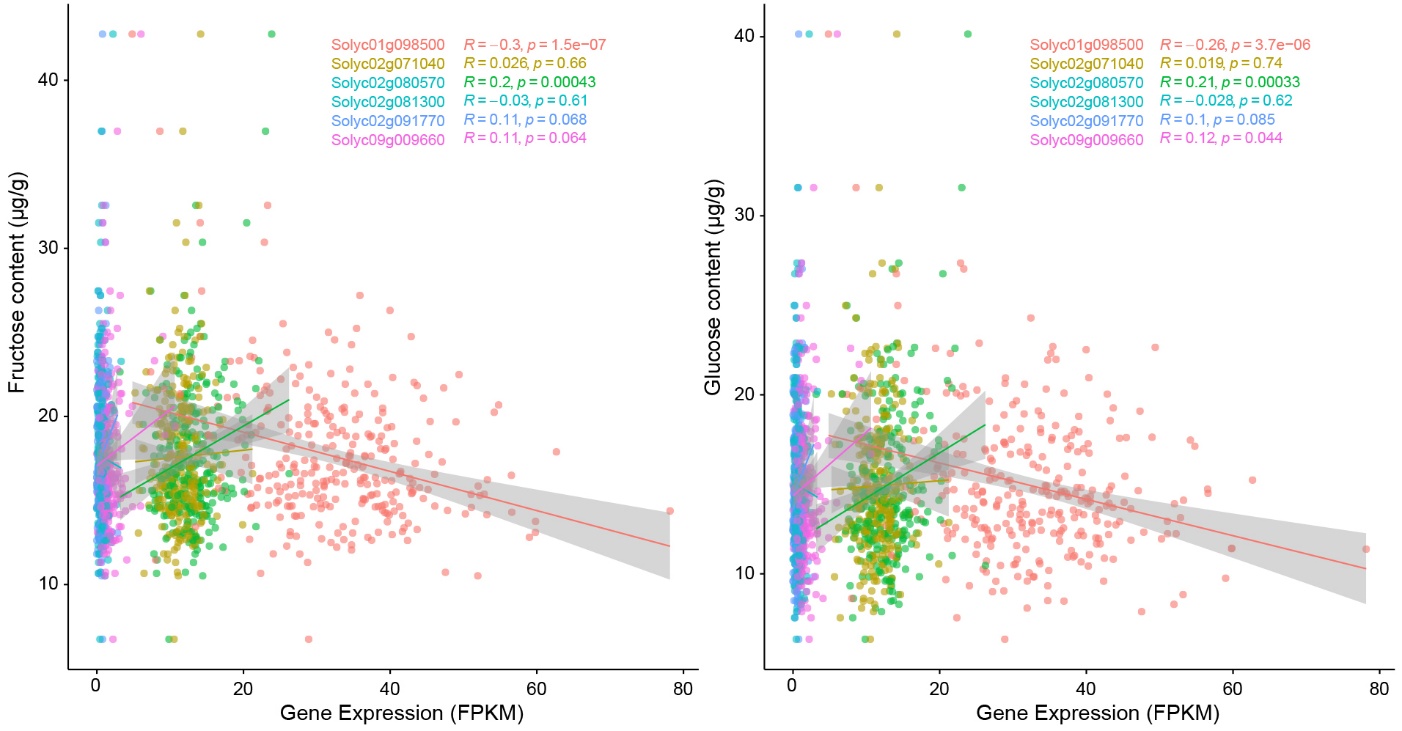


**Supplementary Fig. 12** Correlations between sugar contents (fructose, glucose) and expression of eQTL genes associated with sugars in the natural population.


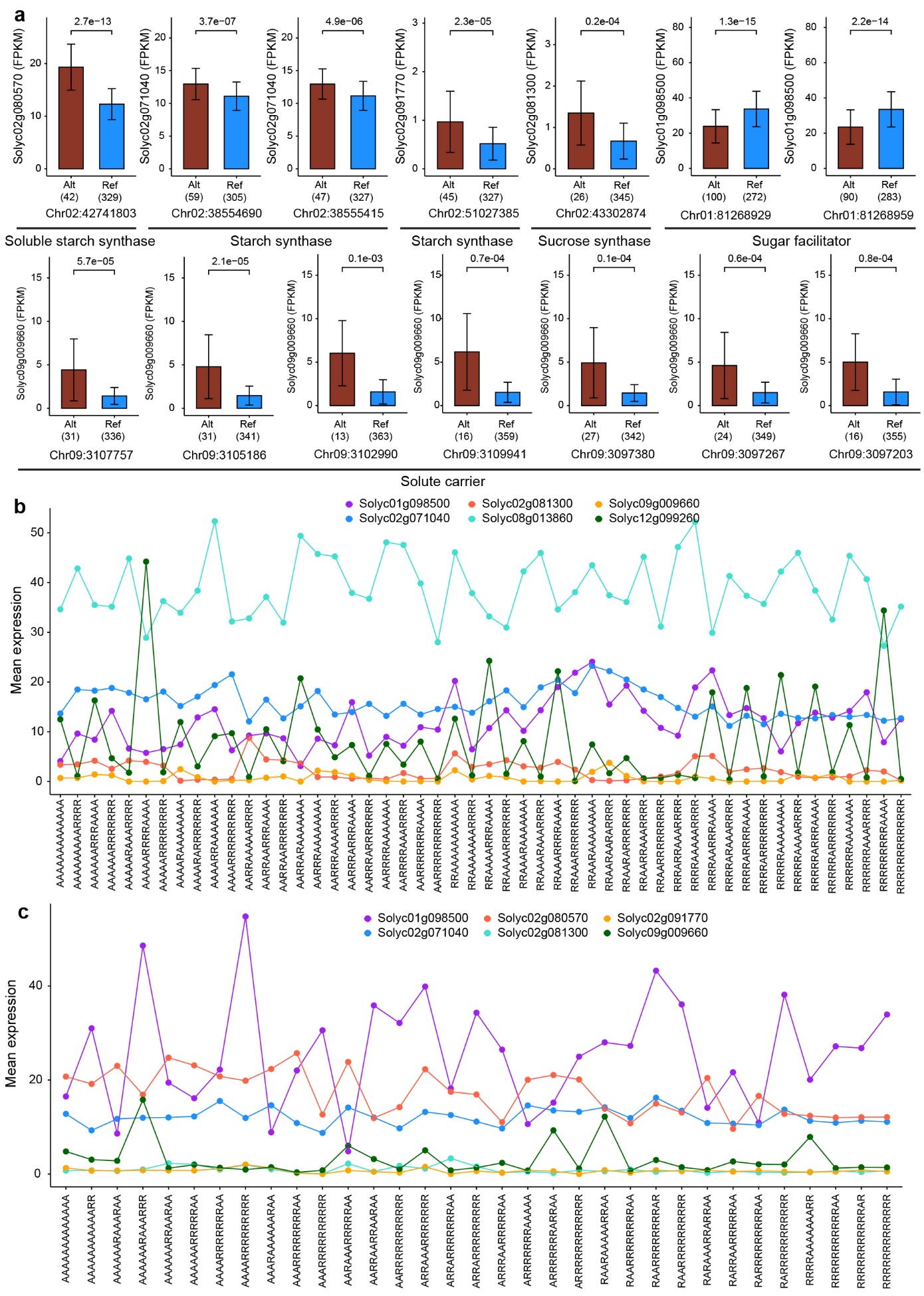


**Supplementary Fig. 13** Expression variation of genes associated with sugars and organic acids in accessions carrying different high-effect SNP alleles. **a**, Expression of sugar and organic acid genes in natural accessions carrying reference (Ref) versus alternative (Alt) alleles at the most significant high-effect SNP loci of the corresponding *cis*-eQTLs. Data are presented in bar plot with mean ± SD. *P*-values were calculated using the Wilcoxon test. Sample sizes (n) are indicated in parentheses below each genotype. **b**,**c,** Expression of sugar and organic acid genes in Pimpi (**b**) and natural (**c**) accessions carrying different haplotypes of the high-effect SNPs. A, alternative allele; R, reference allele. The thirteen high-effect SNPs in the Pimpi population (in order) are: Chr02:38,548,147, Chr02:38,554,690, Chr02:43,302,874, Chr09:3,097,380, Chr01:81,267,611, Chr01:81,268,959, Chr08:3,233,641, Chr08:3,244,090, Chr08:3,244,081, Chr12:65,983,480, Chr12:65,983,488, Chr12:65,983,201, Chr12:65,983,203. The thirteen high-effect SNPs in the natural population (in order) are: Chr02:42,741,803, Chr02:38,554,690, Chr02:38,555,415, Chr02:43,302,874, Chr09:3,107,757, Chr09:3,105,186, Chr09:3,102,990, Chr09:3,109,941, Chr09:3,097,380, Chr09:3,097,267, Chr09:3,097,203, Chr01:81,268,929, Chr01:81,268,959.


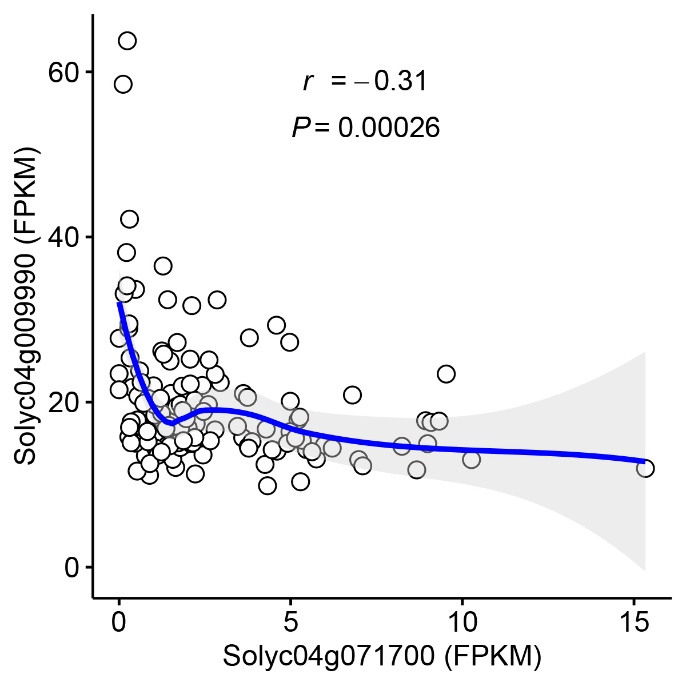


**Supplementary Fig. 14** Expression correlation between *Solyc04g071700* and *Solyc04g009990*. The blue line represents the locally weighted scatterplot smoothing (LOESS) regression curve, illustrating the trend of the relationship, and the gray shaded area indicates the 95% confidence interval of the LOESS fit.
